# Implementation of Selected Assumptions of the Industry 4.0 Concept for the Clothing Sector—The Case of a Personalized Women’s Dress

**DOI:** 10.3390/ma19194236

**Published:** 2026-10-06

**Authors:** Pamela Miśkiewicz, Karolina Serafin

**Affiliations:** Faculty of Textiles and Design, Institute of Architecture of Textiles, Lodz University of Technology, 116 Zeromskiego St., 90-543 Lodz, Poland

**Keywords:** Industry 4.0 concept, sustainable development, women’s dress, thermal insulation properties, CAD/CAM systems, modern sewing machines

## Abstract

**Highlights:**

- The use of fabric made of natural fibers in the clothing sector supports sustainable development and allows for the achievement of appropriate thermal insulation properties for use in clothing intended for use in spring.
- The dress design, form modeling, and virtual prototype sewing were all carried out in the CLO3D program, eliminating the need to sew physical prototypes, which allowed for lower production costs and reduced material consumption, in line with the principles of sustainable development and the Industry 4.0 concept.
- Modern, programmed sewing machines used during the project implementation allow for reduction in the labor intensity of the production process and strengthening of cooperation between humans and machines—in line with the concept of Industry 4.0.
- A women’s dress, designed in accordance with selected Industry 4.0 assumptions, is a personalized and unique clothing product.

**Abstract:**

The contemporary apparel sector requires manufacturers to adapt efficiently to rapidly evolving market trends. Digital technologies and the automation of production processes play a key role in this regard, facilitating increased speed and efficiency in clothing production. Industry 4.0 focuses on sustainable development, digitization, and personalization, integrating human creativity with modern technologies. This article presents the potential application of selected Industry 4.0 principles in the apparel industry, using the example of the design and production of a personalized women’s dress. The project was implemented using natural fiber fabrics, in accordance with the principles of sustainable development, and analyzing their thermal insulation properties. The process used digital garment modeling and virtual sewing in a CLO3D environment, which reduced the number of physical prototypes. The product was manufactured using modern, programmed sewing machines, reducing labor intensity and strengthening human–technology collaboration. It is also worth noting that the women’s dress, developed in accordance with the Industry 4.0 concept, is a product personalized according to the user’s specific needs.

## 1. Introduction

The world of fashion is constantly evolving and changing, shaped by design esthetics, technological advancements, and consumer preferences and expectations. Designers are increasingly eager to embrace digital solutions that enable the expansion and development of creativity. From 3D modeling to virtual fittings, sustainable materials, and AI-powered trend analysis, this development is radically impacting how clothing is perceived and produced [1,2].

The contemporary textile and clothing industry, operating in an intelligent production environment, faces the need to increase efficiency, implement sustainable development principles, and flexibly adapt to dynamically changing market demands. At the same time, maintaining high product quality and meeting the legal requirements that determine its operation and competitiveness are crucial [3,4,5]. Global trends related to sustainable business and the consumer-driven economy model also exert pressure on the sector’s transformation, pushing companies towards greater innovation and environmental responsibility. This stems, among other things, from the growing environmental awareness of customers, who prefer environmentally friendly products and accept their higher prices [3,6,7].

Sustainable development in the virtual clothing sector integrates modern digital technologies with activities focused on environmental and social responsibility. Its goal is to reduce the negative impact of clothing production on the environment, support ethical labor standards, and raise consumer awareness of sustainable decisions while simultaneously leveraging the potential of digital technologies to transform the fashion industry [8,9,10]. In the sustainable virtual production model, digital products and resources are created without physical materials, which allows for reduced resource consumption and material waste. The clothing industry is currently undergoing intensive transformation, resulting from the implementation of modern technological solutions [11,12,13].

Industry 4.0 in the textile sector is a digital transformation involving the integration of intelligent information and communication technologies with design, manufacturing and supply chain management processes, leading to the creation of flexible, autonomous and efficient production systems capable of implementing individualized and sustainable textile production [14,15,16].

With the introduction of Industry 4.0, a number of changes are taking place across the textile industry. Technicians and designers are focusing on: digital fabric and garment design (CAD/CAM and CLO3D); virtual prototyping of clothing products; building and constructing intelligent, communicating machines; automated cutting and sewing; monitoring production parameters using IoT sensors; analyzing production data to improve quality and efficiency; and personalization and more sustainable production, especially with regard to the use of natural resources [14,15,16,17,18].

The development of Industry 4.0 is driven by the increasing digitization of information, communication between devices, and the virtual representation of production systems. In the smart factories of the future, repetitive tasks previously performed by humans will gradually be replaced by automation and robotics solutions. These changes contribute to increased productivity, improved work environment standards, and the creation of new professions [14,19,20,21].

Transforming production processes requires the implementation of advanced technologies, including computer-aided testing of yarn parameters, fabric modeling and simulation, as well as intelligent machines capable of monitoring their own technical condition to prevent failures. Key solutions also include automatic cutting and sewing systems and digitally controlled knitting technologies [14,22].

This article presents the potential application of selected Industry 4.0 principles in the clothing sector, using the example of the design and production of a personalized women’s dress. The work involved exclusively using fabrics made of natural fibers. To support sustainable development and digitization, the dress was constructed and virtu-ally sewn using CLO3D software to reduce the number of physical prototypes. In the final stage, including the dress’s production, modern, digitally controlled sewing machines were used, requiring prior programming, reducing labor input and intensifying human interaction with technological solutions. Additionally, selected natural fiber fabrics were analyzed for their thermal insulation properties to assess the feasibility of using the designed product in spring. It should be emphasized that the developed clothing product, in line with Industry 4.0 principles, is characterized by a high degree of digitization, sustainable development, personalization, and uniqueness.

The uniqueness of the article results from the combination of the analysis of thermal insulation properties of textile materials with modern tools for digital clothing design (CLO3D) and the automation of production processes.

In this article, the authors try to find the answer to the following question: What are the benefits and limitations of using Industry 4.0 tools in the process of designing and manufacturing a personalized women’s dress?

## 2. Materials

Fabrics made from natural fibers were selected for the women’s dress: linen, cotton, hemp, and ramie (Table 1). Two variants of the linen fabric were used.

The natural fiber fabrics selected for the women’s dress differed in their raw material composition, weave, weight per unit area, and thickness. Only the linen fabric used as the base of the dress did not contain 100% linen—but 85%. Three of the used fabrics had a plain weave—linen, hemp, and cotton—while the other two—ramie and the linen base—had a twill weave. The highest masses per unit area were achieved by ramie (246 g/m^2^) and the linen base (241 g/m^2^). The remaining natural fiber fabrics ranged in mass per unit area from 123 to 168 g/m^2^. In terms of thickness, the cotton fabric was the thinnest, at 0.34 mm, while the linen base (0.63 mm) and ramie (0.59 mm) were the thickest.

## 3. Methods

### 3.1. Air Permeability

Air permeability, also known as breathability, is a characteristic of clothing that influences the microclimate under clothing during use. Breathability significantly affects the thermal insulation properties of the product [23,24].

Air permeability testing should be performed according to the PN-EN ISO 9237 standard [25]. According to the standard, air permeability is measured by the velocity of air passing perpendicularly through the tested material sample under conditions determined by its surface area, pressure difference, and time [23,24]. According to the standard, samples should be acclimatized for 24 h at an air temperature of 20 °C and a relative air humidity of 65%. The test is performed on an air permeability tester, and the minimum sample size required for measurement is 20 cm × 20 cm.

In accordance with the PN-EN ISO 9237:1998 standard, all fabrics used in the women’s dress design were acclimatized prior to measurement. Air permeability tests were performed on fabrics measuring 50 × 50 cm, and 10 measurements were taken for each fabric. The surface area of the test sample was 20 cm^2^, and the pressure difference was 100 Pa.

Depending on the obtained measurement result, the tested textile materials can be classified as permeable materials when their air permeability is greater than 600 mm/s, and medium-permeability materials include those with a permeability range of 300–600 mm/s, while low-permeability materials are characterized by air permeability below 300 mm/s.

### 3.2. Thermal Insulation Properties—Alambeta Device

To test the thermal insulation parameters of selected fabrics, an Alambeta device from Sensora was used. This measuring device is used to evaluate the thermophysical parameters of materials in various states—static and dynamic. This device is classified as a plate measurement method. Its operation is based on the phenomenon of thermal convection; heat generated by a heated upper plate (plate temperature up to 32 °C) passes through the tested textile material to the cold lower plate (normal climate temperature of 25 °C), which is in contact with it. The pressure force between the plates is 200 Pa [26,27]. The device allows for the measurement of the seven indicators described below.

Thermal conductivity (λ) is defined as the ability of a material to conduct heat and is expressed in units of Wm^−1^K^−1^. It is described as the amount of heat transferred per unit of surface area perpendicular to the direction of the heat flux, per unit of time and temperature gradient.

Thermal resistance (R) is the resistance of a material to heat exchange due to the temperature difference, expressed in units of W^−1^Km^2^.

Thermal absorption (b), defined by the heat absorption coefficient, characterizes the tested material sample in terms of its warm or cold sensation upon contact, and its unit is Wm^−2^s^1/2^K^−1^. Higher values of this parameter indicate that the material is colder upon contact with the skin, while lower values of thermal absorption mean that the material is perceived as warmer to the touch.

Thermal diffusivity (a) is a physical quantity characterizing transient heat conduction, expressed in m^2^s^−1^. This parameter determines the rate of response of the tested material to temperature changes.

The maximum-to-stationary heat flow ratio is defined as the ratio of the maximum heat flow when a textile material touches a warmer surface to the stationary heat flow through the material after flow conditions stabilize. This parameter determines the material’s warm or cold sensation upon contact.

Maximum heat flow density at the point of contact is the amount of heat conducted through a unit of material surface area per unit of time. This parameter is expressed in Wm^−2^.

Additionally, the Alambeta device measures the sample thickness in mm [26,27,28].

### 3.3. CLO3D Program

The construction process is a key element in clothing design and manufacturing, as it allows for the transformation of a sketch into a set of components that, through sewing, create a finished product. Modern 3D CAD systems used in clothing design and construction enable virtual fittings and the simulation of the appearance of woven and knitted fabrics. Using these systems, it is possible to sew two-dimensional garment forms onto a digital avatar, allowing for the visualization of a garment prototype [29,30,31].

**Figure 1:**
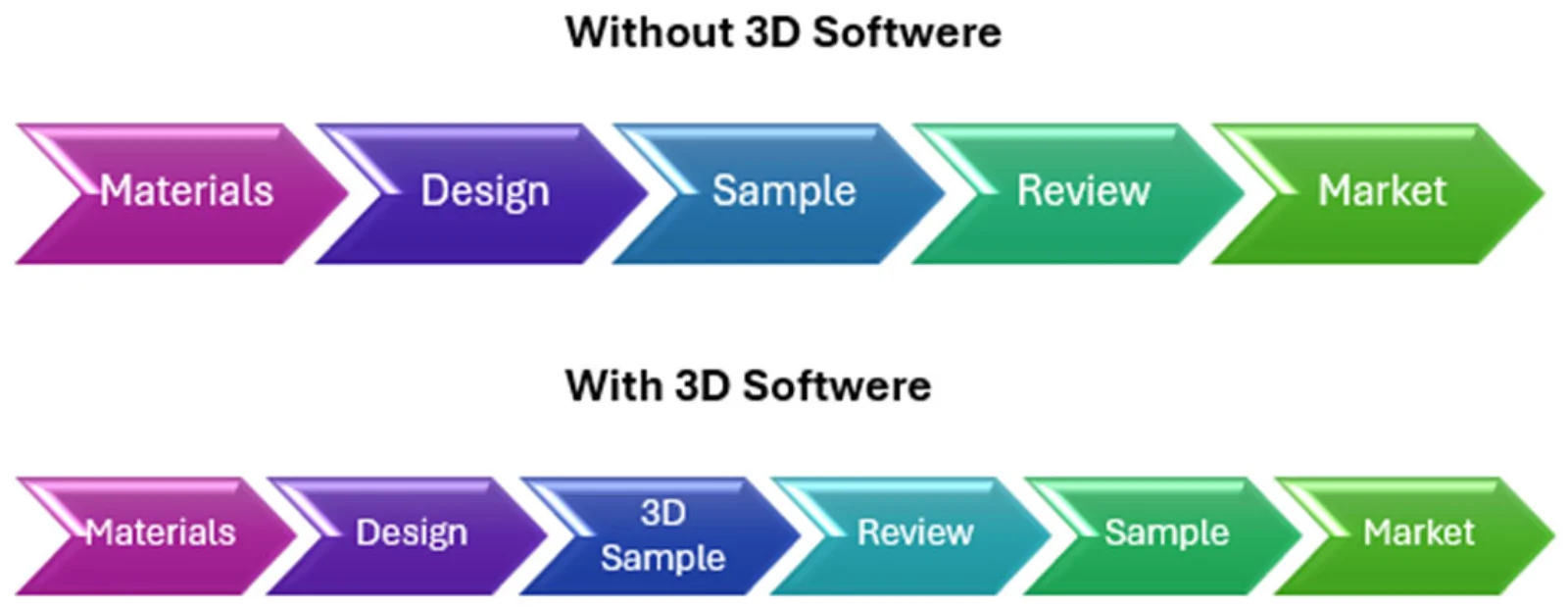
Comparison of the clothing design process with and without 3D software.

CLO3D is advanced CAD software for 3D clothing design and simulation. With the ability to create realistic digital clothing prototypes, users can significantly shorten production time by reducing the number of physical prototypes, which reduces material consumption and accelerates the entire production process (Figure 1). CLO3D supports the circular economy and waste reduction [32,33].

The program’s key feature is the ability to create clothing structures in two-dimensional (2D) space, then transform them into forms that can be immediately visualized in a three-dimensional (3D) model. Created clothing elements can be directly visualized on the avatar, allowing for a realistic presentation of the clothing design in motion and space. The user can manipulate the garment’s form and its adaptation to the avatar’s silhouette in both 2D and 3D. The program allows for editing of material properties such as thickness, stiffness, and proper fit, enabling a realistic representation of fabric behavior on the human body.

The software offers a wide range of tools, including interactive editing of garment forms, allowing for precise modification of patterns, fine-tuning of details, and easy adjustments. The program also features a virtual sewing function, which allows users to simulate the process of joining materials and verify the final appearance of the garment.

The program offers pre-made avatars categorized by gender, age, and overall size, which can be edited for proportions and dimensions. This allows the program to create an avatar that matches the user’s individual dimensions. Furthermore, the user can animate the avatar’s movements, allowing for the evaluation of clothing behavior during movement [32,33].

### 3.4. Modern Sewing Machines

Automation has played a key role in industrial development for years, enabling greater precision and repeatability, which directly translate into higher quality and accuracy in production processes. In the textile industry, robotic solutions are used to reduce labor-intensive technological operations, while in modern times, automation remains closely integrated with computer-assisted robotics, contributing to increased efficiency and productivity of production processes.

The following machines were used to make the designed women’s dress:

- A PFAFF 3511 automatic sewing machine for small elements;
- An ASS BASS 3500 TJ automatic cut pocket machine;
- A Pfaff 3843 sleeve sewing machine;
- A JUKI MF-7723-U10-B56 flatbed renderer;
- A Pfaff 1163 lockstitch machine with binder;
- An Impall JAPSEW 781X hand-stitch-imitating machine;
- A Dürkopp Adler 581-151 buttonhole machine;
- A PFAFF 3307 button sewing machine.

## 4. Results

### 4.1. Air Permeability Test Results of Selected Natural Fiber Fabrics

**Figure 2:**
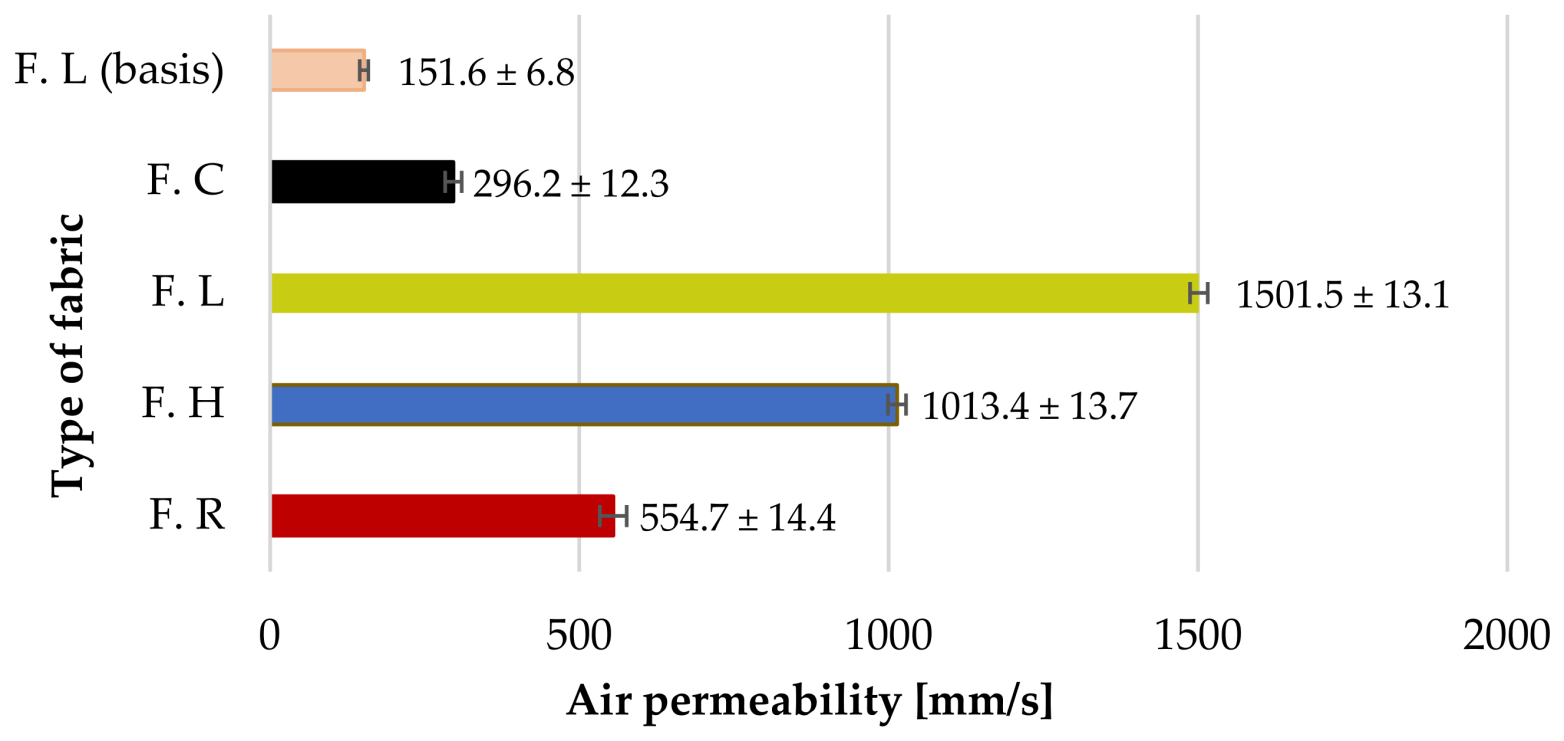
Air permeability chart of natural fiber fabrics selected for a women’s dress.

The air permeability test was performed in accordance with the PN-EN ISO 9237 standard [29] using a Model FX 3300 air permeability tester from Textest Instruments (Switzerland). The obtained air permeability results for natural fiber fabrics are presented in Figure 2.

In order to facilitate the descriptive analysis of the test results for air permeability and thermal insulation properties of individual fabrics, the following simplifications were adopted:

The linen fabric used as the base of the dress is marked as F. L (basic);

The cotton fabric is marked as F. C;

The linen fabric is marked as F. L;

The hemp fabric is marked as F. H;

The ramie fabric is marked as F. R.

The colors of the bars in the charts are related to the color that a given natural fiber fabric presents on the designed product, as shown in Figure 9.

The highest values of the tested parameter (Figure 2) were recorded for linen fabric (1501.5 mm/s) and hemp fabric (1013.4 ms^−1^) which are classified as permeable materials characterized by high porosity. The lowest air permeability results were obtained for linen fabric (base) at 151.6 mm/s and cotton fabric at 296.2 mm/s; therefore, these are poorly permeable materials with low porosity. Furthermore, these fabrics are densely woven, as can be observed in the microscopic images provided in Table 1.

### 4.2. Test Results of Thermal Insulation Properties of Selected Natural Fiber Fabrics

**Figure 3:**
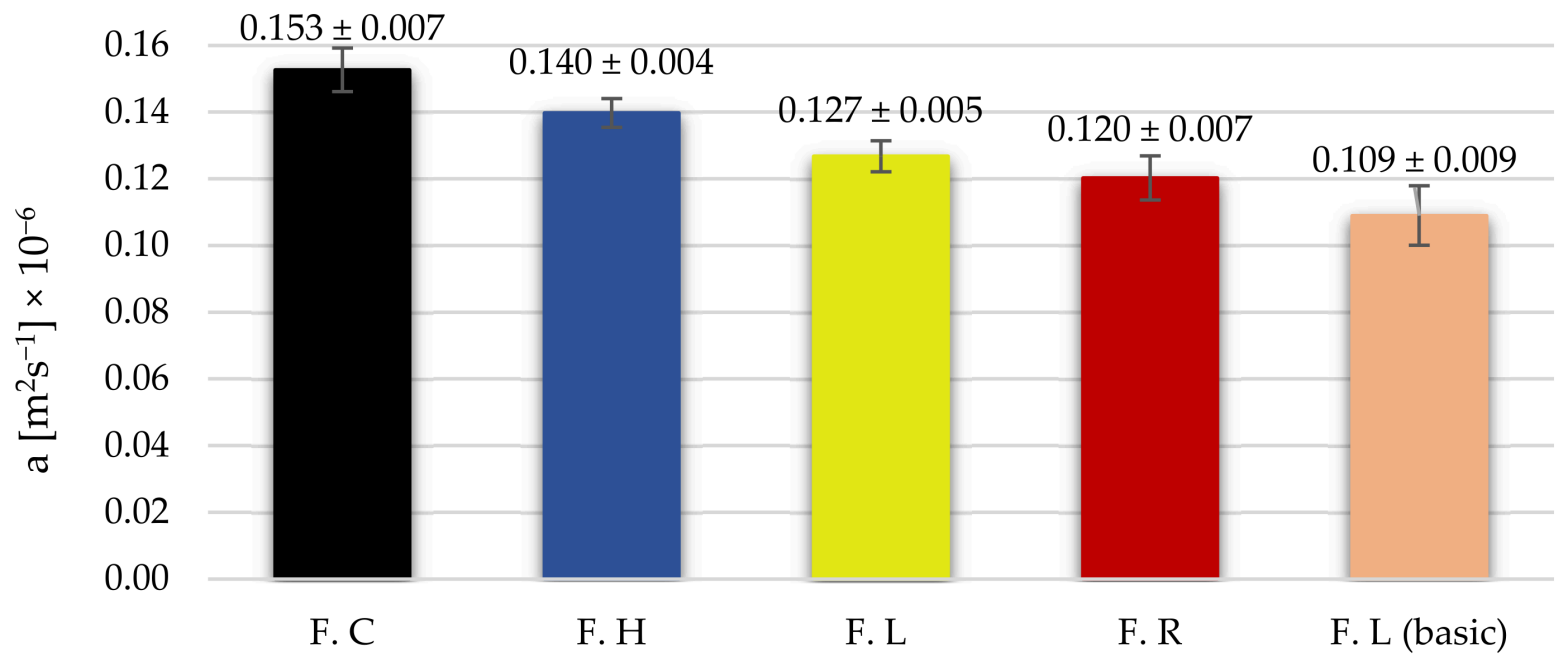
Thermal diffusivity results of fabrics selected for the production of a women’s dress.

**Figure 4:**
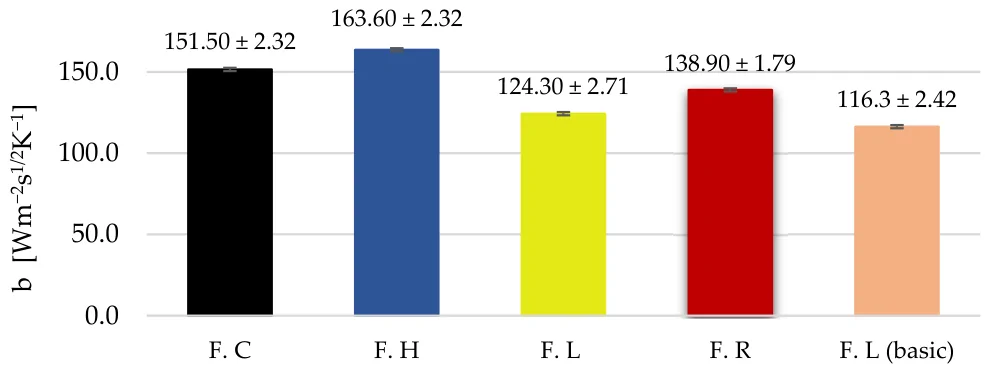
Thermal absorption results of fabrics selected for the production of a women’s dress.

**Figure 5:**
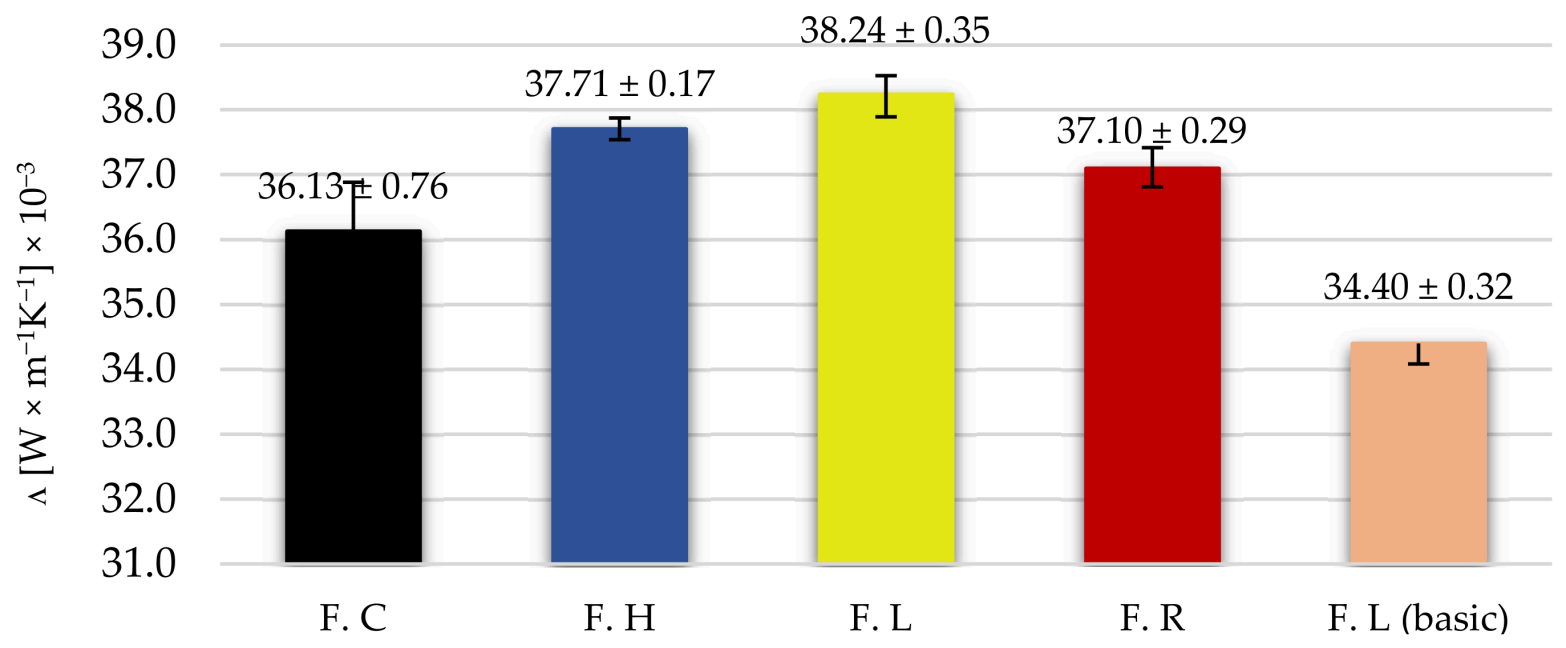
Thermal conductivity results of fabrics selected for the production of a women’s dress.

**Figure 6:**
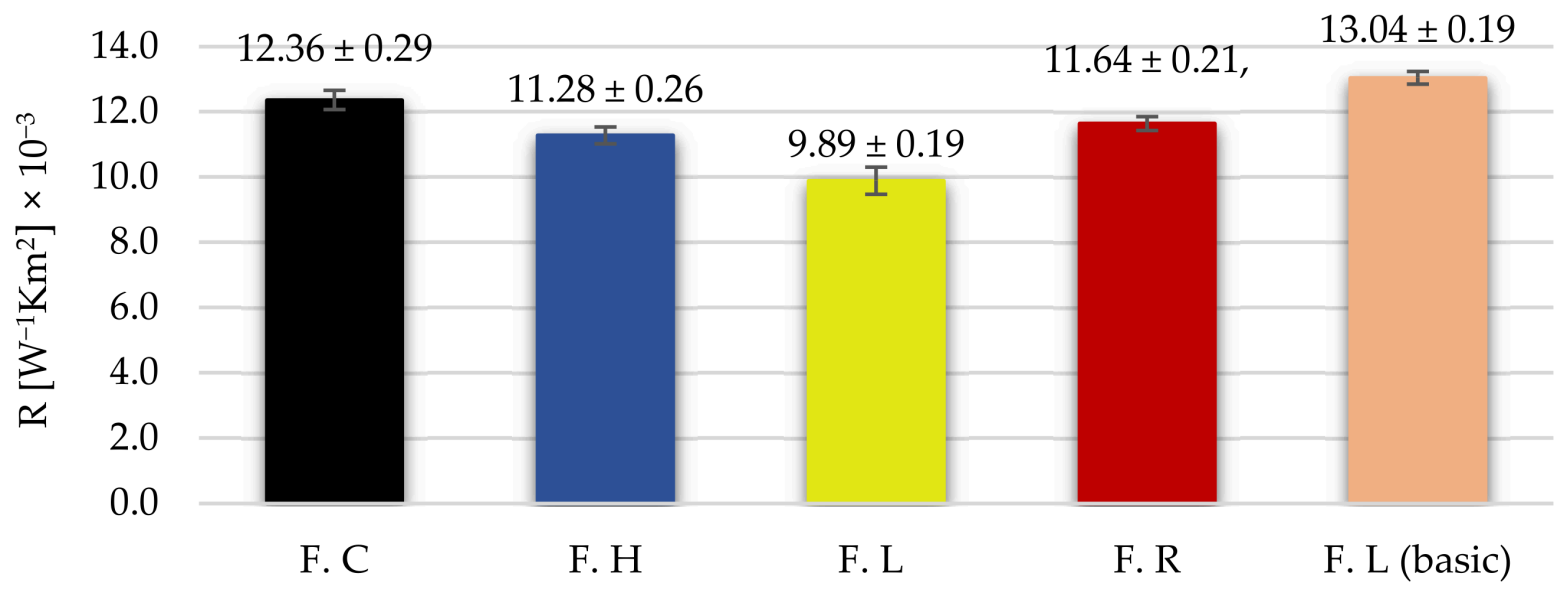
Thermal resistance results of fabrics selected for the production of a women’s dress.

**Figure 7:**
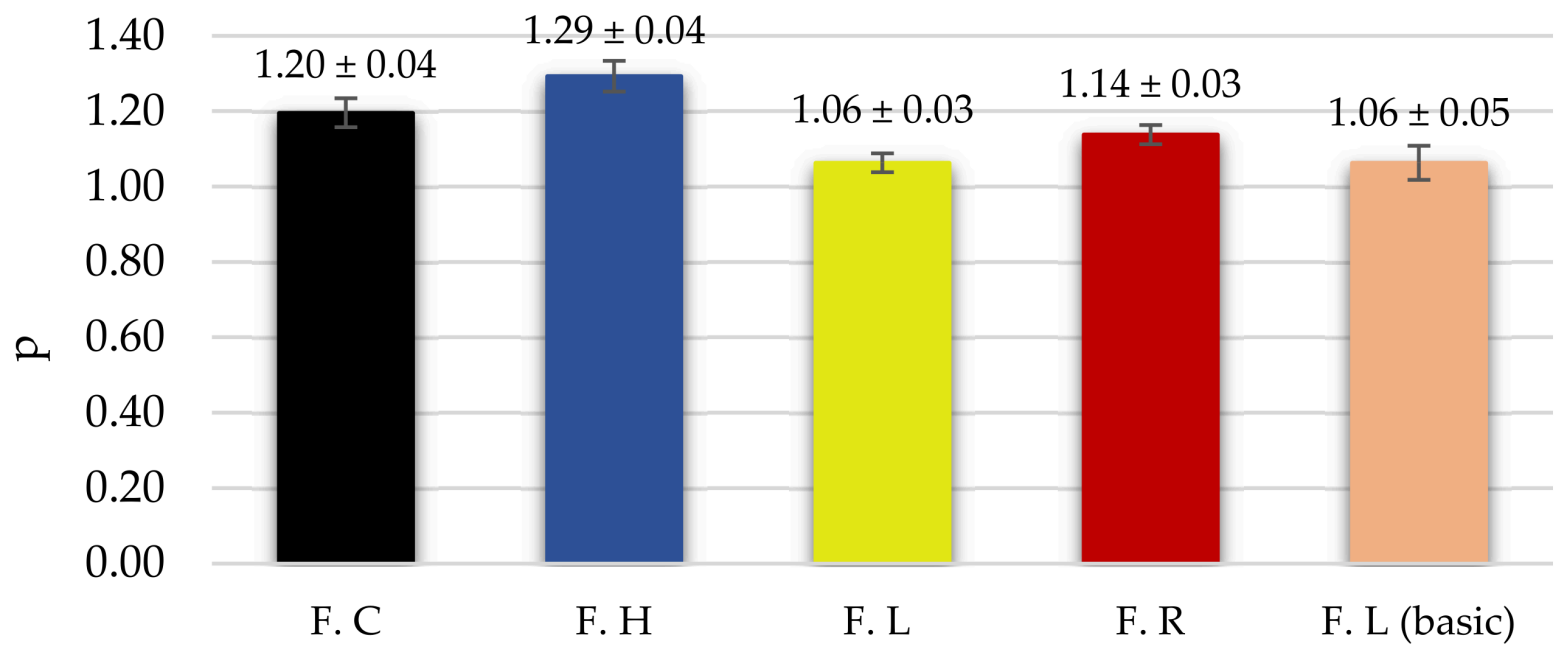
Results of the maximum-to-stationary heat flow ratio of the fabrics selected for the production of a women’s dress.

**Figure 8:**
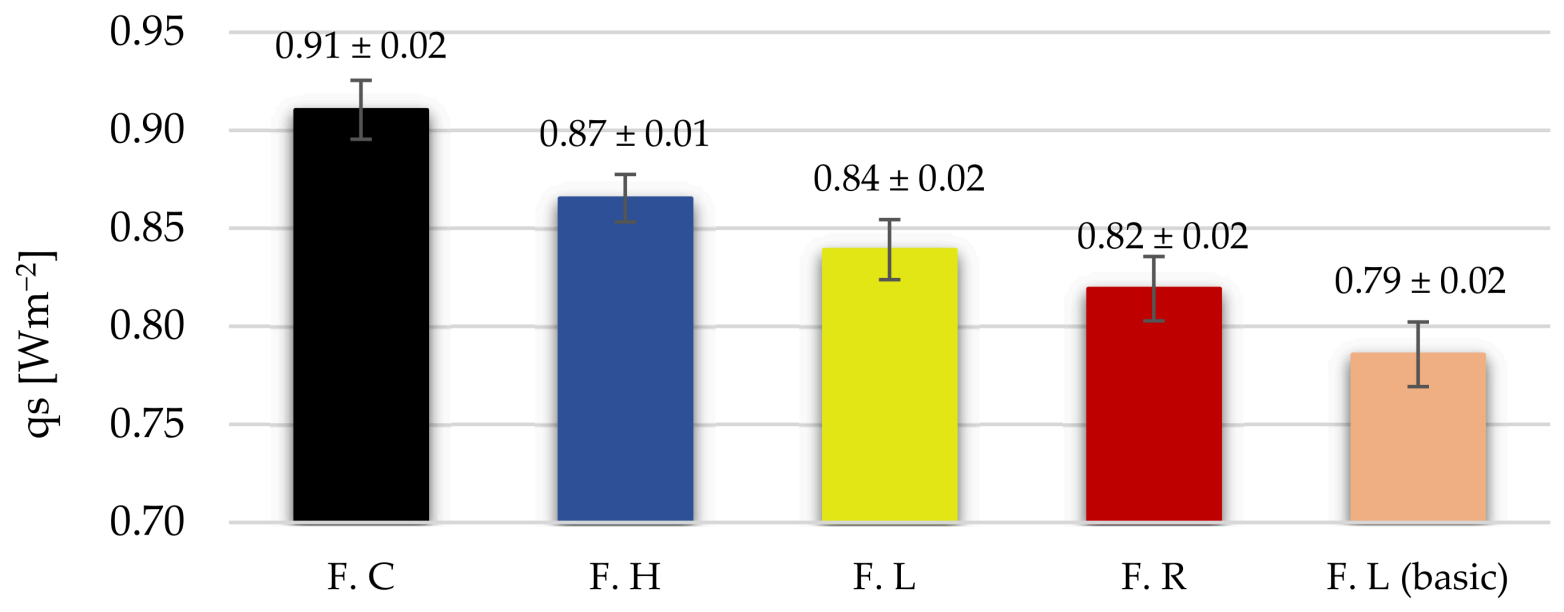
Results of the maximum heat flux density of fabrics selected for the production of a women’s dress.

Thermal insulation properties were tested using an Alambeta device. Ten measurements were taken for each type of fabric selected for the women’s dress. A single test allowed results to be obtained for seven parameters defining the thermal insulation properties of the selected fabrics. The results are presented in the Figure 3, Figure 4, Figure 5, Figure 6, Figure 7 and Figure 8.

Cotton fabric has the highest thermal diffusivity value (Figure 3) of 0.153 × 10^−6^ m^2^s^−1^, meaning it reacts most quickly to temperature changes and will conduct heat quickly. Linen fabric (base) showed the lowest value of the tested parameter, i.e., 0.109 × 10^−6^ m^2^s^1^, indicating that it was slowest to respond to any temperature changes.

The highest values of the tested parameter (Figure 4) were obtained for hemp fabrics (163.6 Wm^−2^s^1/2^K^−1^) and cotton (151.5 Wm^−2^s^1/2^K^−1^), meaning they are the coldest in contact with the skin surface. Linen fabrics showed the lowest thermal absorbency value, meaning they are the warmest in contact with the skin among all the tested fabrics.

Thermal conductivity is a parameter that determines a material’s ability to conduct heat. It depends primarily on the material’s structure and porosity. Therefore, under identical conditions, significantly more heat will flow through a material with a higher thermal conductivity coefficient. The lowest thermal conductivity value (Figure 5) was obtained for linen fabric (base), at 34.40 × 10^−3^ Wm^−1^K^−1^, meaning that the least heat will flow through it. This is due to its densely woven fabric containing the smallest pores in its structure. The highest value of the tested parameter was recorded for linen fabric, at 38.24 × 10^−3^ Wm^−1^K^−1^, indicating its high porosity.

According to the results presented in Figure 6, the highest thermal resistance value was recorded for the linen (base) fabric (13.04 × 10^−3^ W^−1^Km^2^), as it is characterized by the highest mass per unit area and thickness, as well as low porosity. As the thickness of the tested fabric increases, the thermal resistance value increases, which is associated with better protection against cold. The lowest value of the tested parameter was observed for the linen fabric (9.89 × 10^−3^ W^−1^Km^2^), meaning that it offers the least resistance to heat flow due to its high porosity.

The maximum-to-stationary heat flow ratio, similarly to thermal absorptivity, determines the sensation (hot/cold) of a fabric in contact with the skin. The highest value of this parameter (Figure 7) was observed for hemp fabric, making it the coolest to the touch of all the tested fabrics. The lowest value of the maximum-to-stationary heat flow ratio was obtained for linen fabrics, making them the warmest to the touch.

The highest value of the tested parameter of 0.91 Wm^−2^ was recorded for cotton fabric; therefore, it is characterized by the maximum heat flux density at the contact point (Figure 8). Among all tested fabrics, the lowest value of the maximum heat flux density was recorded for linen (base) fabric—0.79 Wm^−2^.

### 4.3. Dress Construction and Modeling in CLO3D

This shift dress (a dress with a straight cut—no waistline and a loosely flowing skirt) is inspired by the style of the 1960s and the designs of Yves Saint Laurent, particularly his collection inspired by the paintings of Piet Mondrian. This women’s dress combines minimalist form with artistic expression. The dress’s geometric division and bold colors, such as red, navy blue, yellow, black, and beige, create a dynamic composition. The contrasting collar and three-button front closure emphasize the spirit of the 1960s. The mini length was one of the defining details of this style, as dresses became shorter and more relaxed at the time, allowing women of the era to express greater independence through their clothing. The slightly flared cut; flap pockets; and long, slightly flared sleeves with drawstrings add a touch of lightness to this women’s dress. During the design phase of the dress, fabrics made of natural fibers were selected: linen, cotton, hemp and ramie; however, the linen that formed the basis of the dress was a mixture of: linen (85%), polyester (10%) and elastane (5%) because it was assumed that the dress would be worn in spring. The dress was personalized for a woman of size S: bust, 84 cm; waist, 66 cm; hips, 92 cm; and height, 156 cm. Three cm of clothing allowance was added to the templates. A size S avatar from the avatar library was used in the CLO3D program. After determining the product’s fit, the dress was tried on for a user with the following measurements: bust, 84 cm; waist, 66 cm; hips, 92 cm; and height, 156 cm. The user’s subjective assessment determined the fit was appropriate, as it did not restrict movement, the sleeves and length were appropriate, and the dress fit the wearer’s figure appropriately.

**Figure 9:**
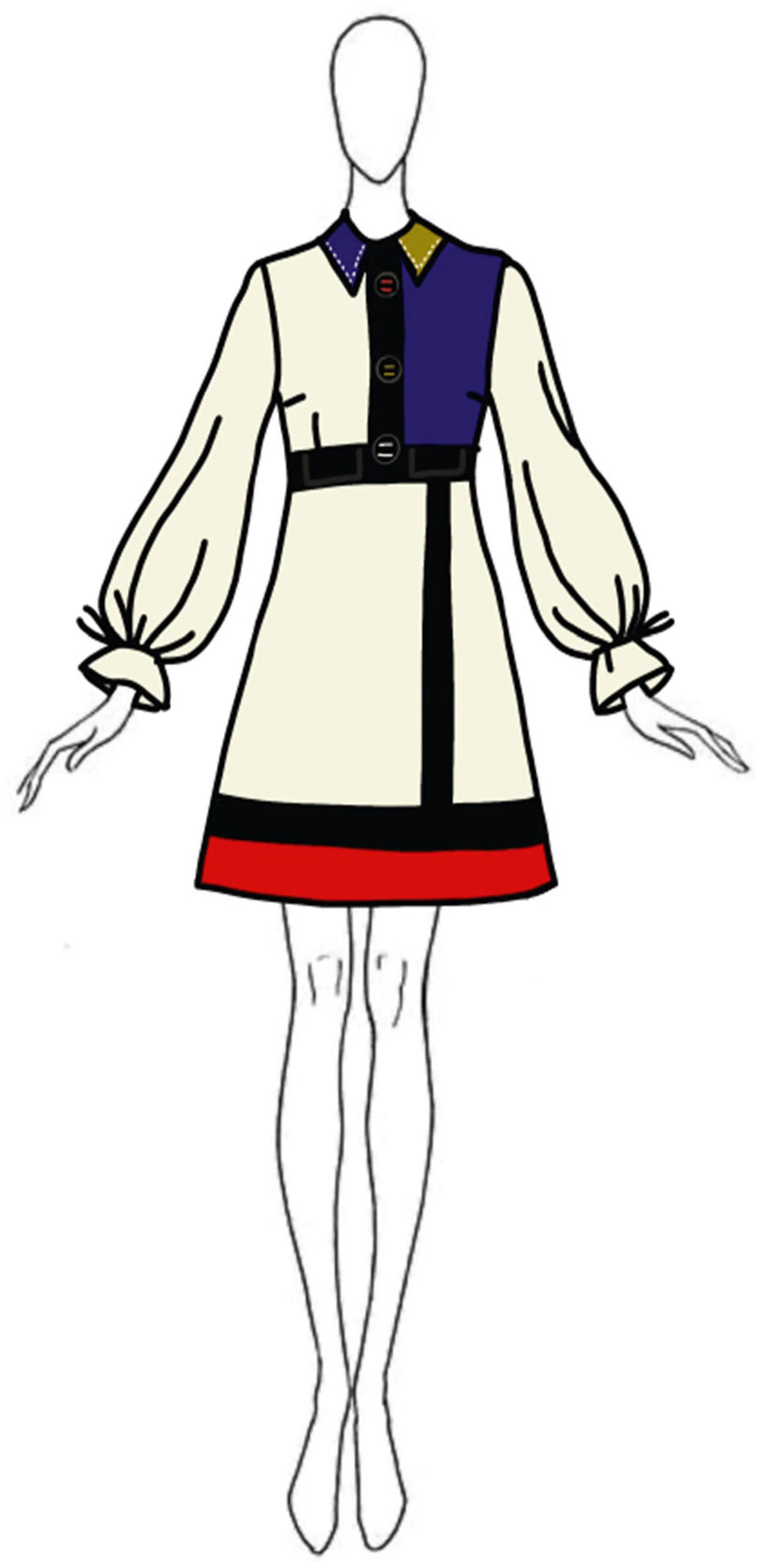
The 1960s-inspired dress design drawing made in CorelDRAW.

The journal illustration below (Figure 9) presents a design for a women’s dress inspired by the 1960s, exemplifying the application of various Industry 4.0 principles to the textile sector.

**Figure 10:**
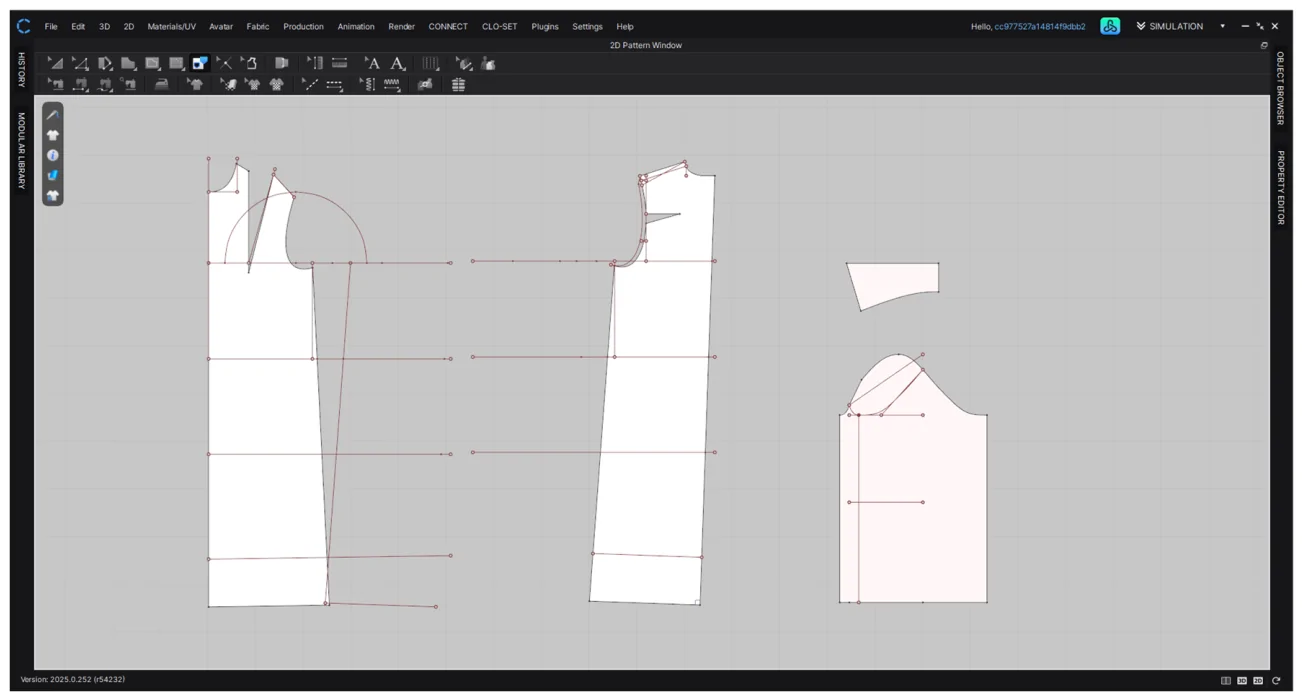
Extracting templates using the “Trace” function.

The design, modeling, and templates for the shift dress were created in CLO3D. Before constructing the dress, the avatar had to be adjusted to the specific dimensions for which the design was made. The next step was to create the construction mesh for the women’s dress, sleeves, and collar in CLO3D. First, a rectangle was drawn to form the construction mesh. The next step involved modeling the dress—a process that involves transforming the construction lines to approximate the body shape. In the program, this involves changing selected straight lines to curved (rounded) lines (Figure 10).

**Figure 11:**
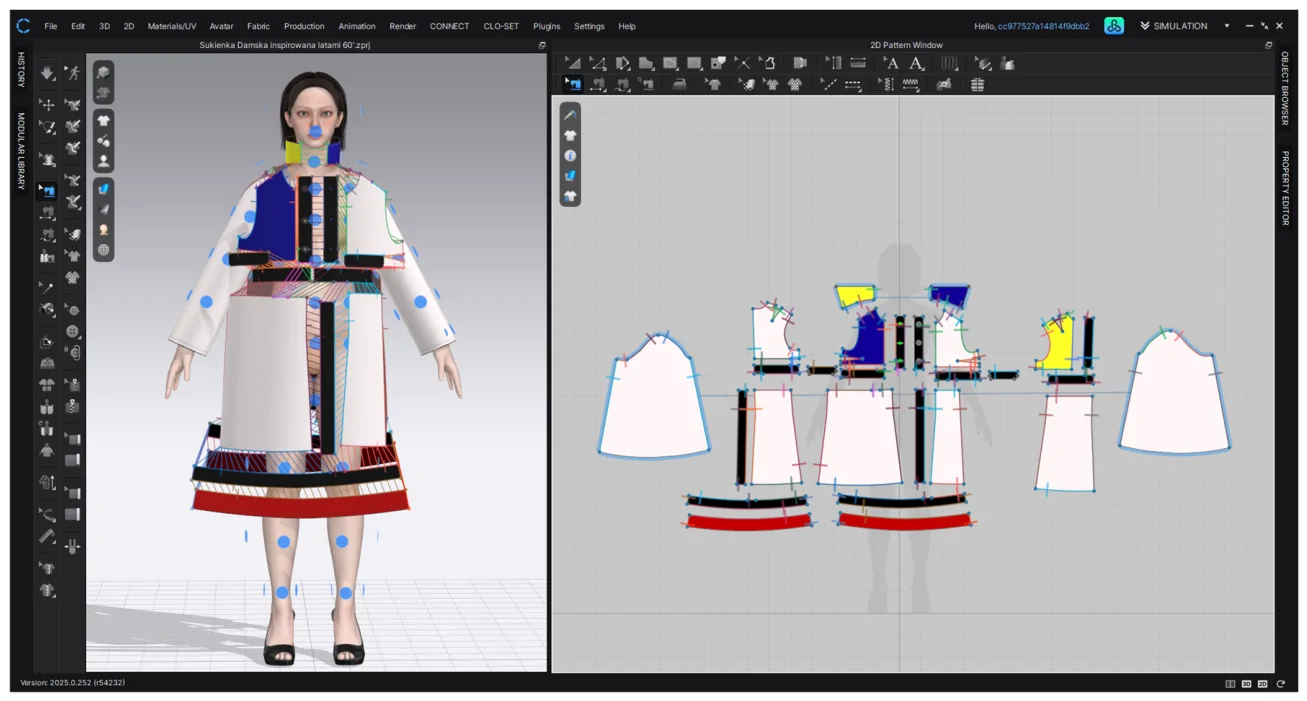
Arrangement of dress templates on the avatar with visible sewing lines.

Further modifications to the patterns included relocating the darts, widening the sleeves, modifying the dress patterns, and checking the shoulder lines between the front and back. The next step involved virtual sewing, which involved arranging the dress forms on the avatar, then adjusting their positions on the avatar’s figure (Figure 11). Colored pairs of lines indicate which dress elements were joined together. Buttons and buttonholes were also added, and the dress fabric was selected to closely match the fabrics selected for the physical prototype.

**Figure 12:**
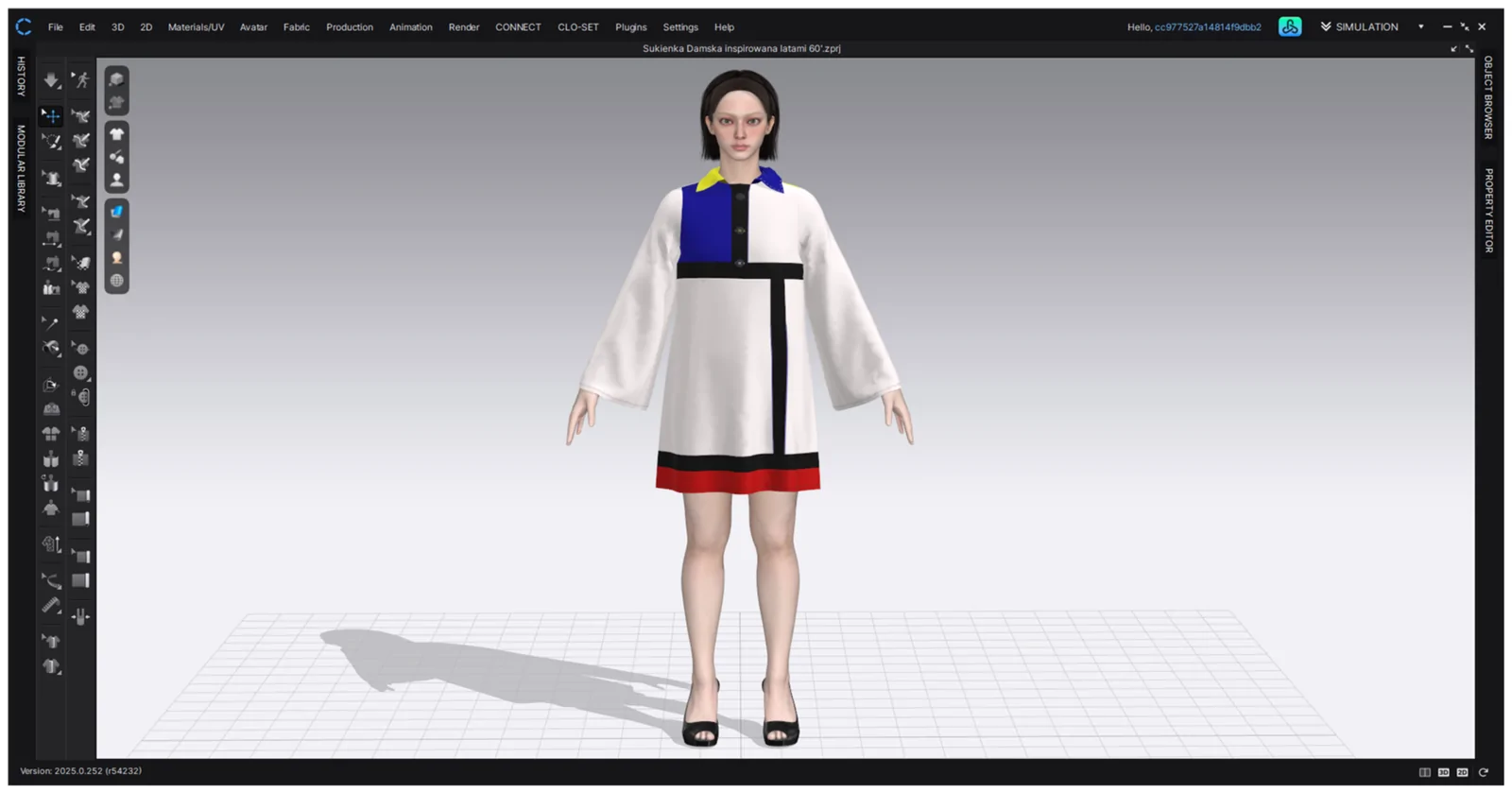
Virtual fitting of a women’s dress—front.

The virtually sewn designed women’s dress is shown in Figure 12.

Using CLO3D significantly shortened design preparation time compared to drawing on paper and streamlined corrections. This type of software reduces the number of physical prototypes, allows for faster error correction, lowers production costs, and reduces material consumption and waste, consistent with the Industry 4.0 concept. Furthermore, the program allows for editing of material properties, color, and pattern.

### 4.4. Characteristics of Modern Sewing Machines Used to Create the Designed Women’s Dress

- PFAFF 3511 automatic sewing machine for small elements

**Figure 13:**
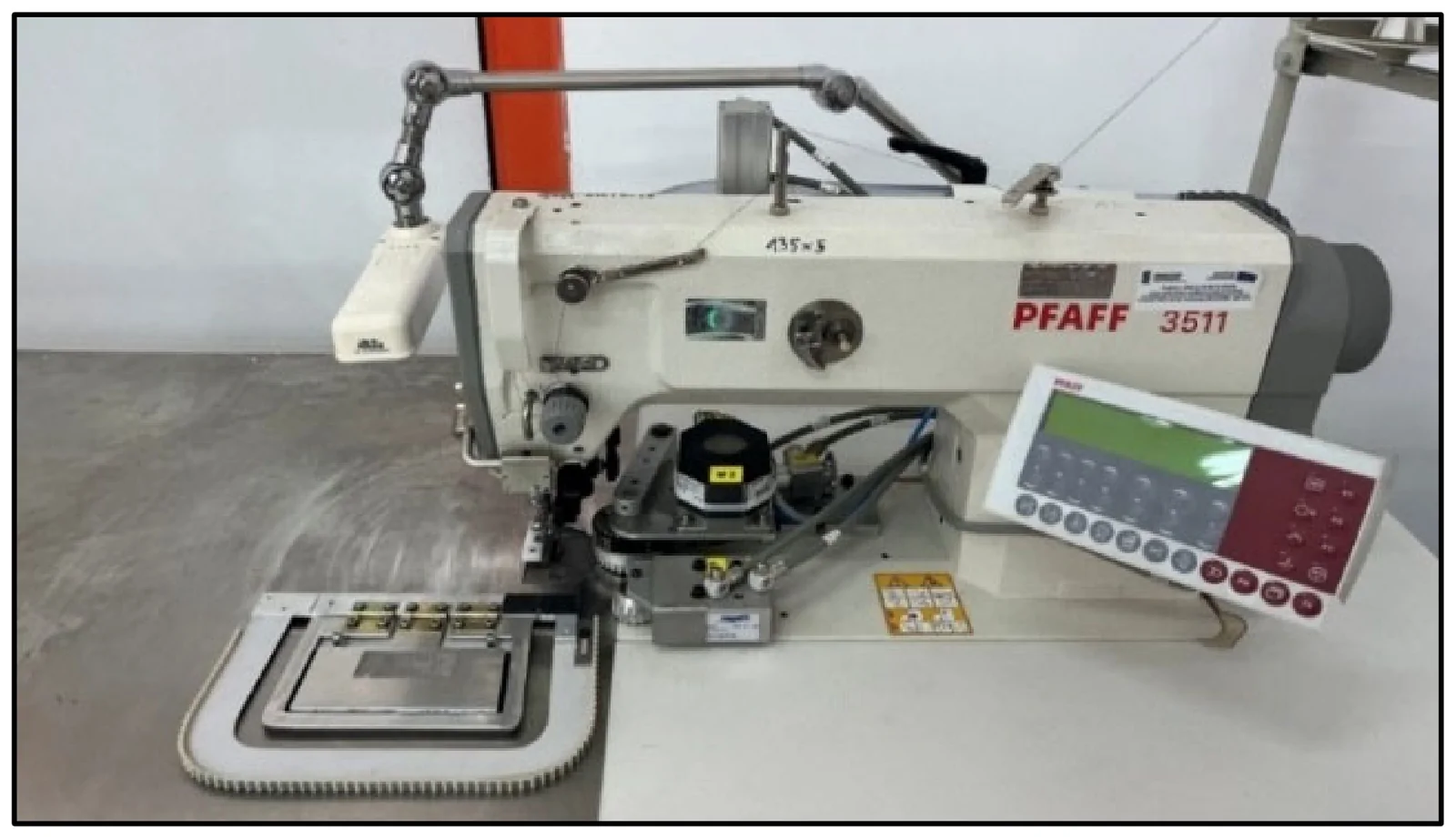
Automatic sewing machine for small elements.

**Figure 14:**
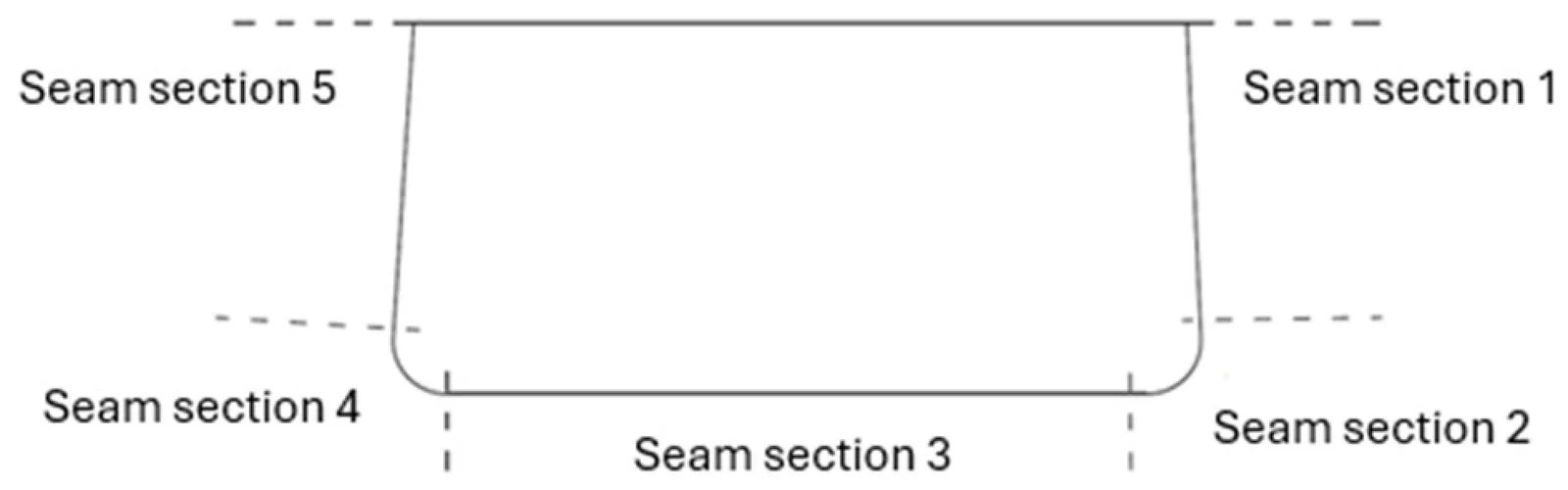
Programming the sewing process of a flap on a machine for sewing small elements, divided into sections.

The PFAFF 3511 automatic sewing machine for small elements (Figure 13) uses a shuttle stitch and is used to sew pocket flaps, collars, and cuffs. The machine is equipped with cassettes into which the material is placed to obtain a properly sewn flap. The operator’s role is limited to properly arranging the material in the cassette, then placing it in the machine’s workspace and pressing the pedal to initiate the sewing process [34]. Settings for five sections were programmed on the machine to enable the flap sewing process. The division of the flap into individual sections is shown in Figure 14.

- ASS BASS 3500 TJ Automatic cut pocket machine

**Figure 15:**
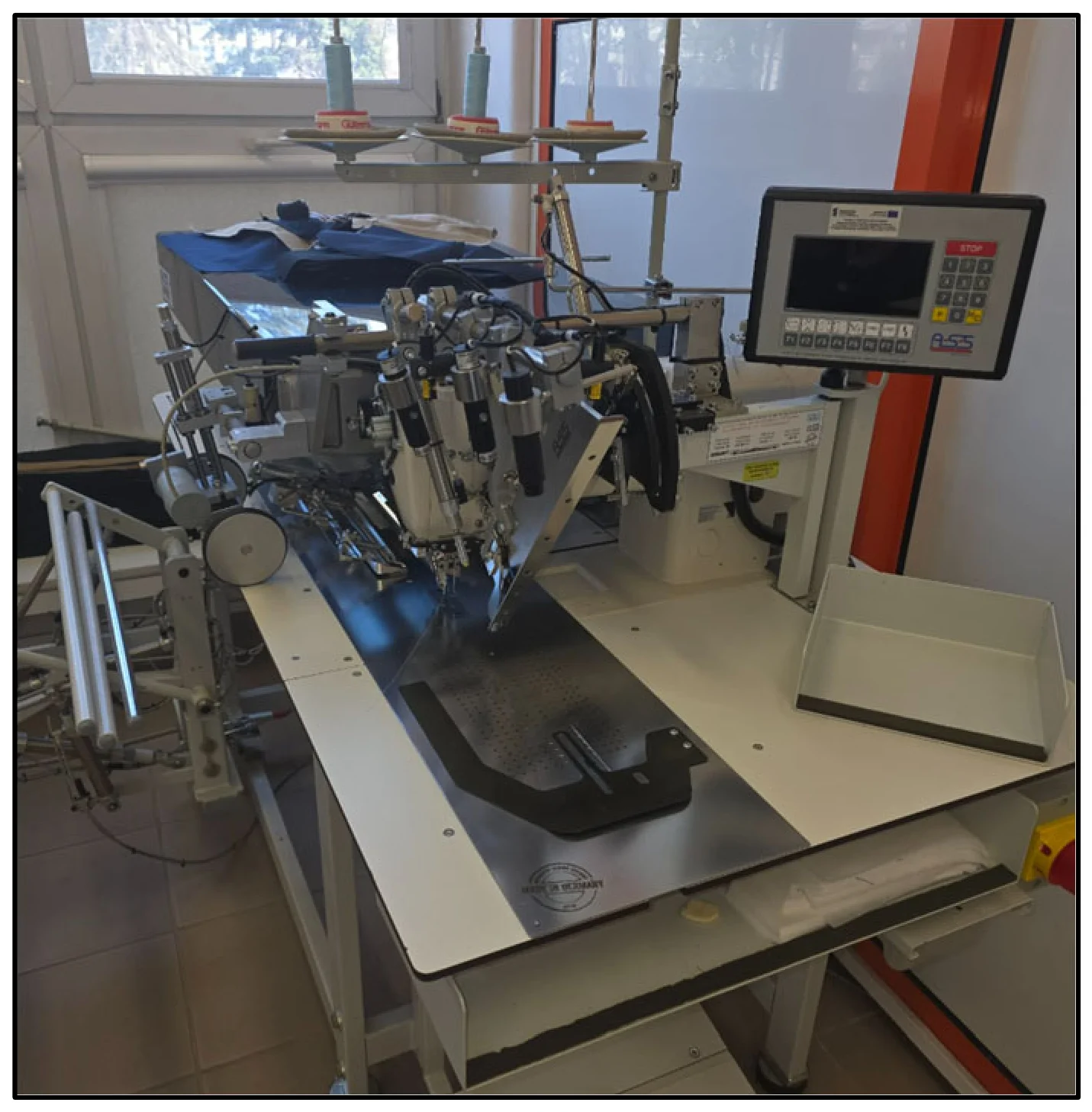
ASS BASS 3500 TJ automatic cut pocket machine.

The ASS BASS 3500 TJ automatic pocket sewing machine (Figure 15) is a lockstitch sewing machine designed for sewing straight and angled pockets, with the option of sewing pockets with one or two welts. The machine’s core is a machine head that, without additional equipment, can function as a two-needle lockstitch machine with the option of switching off the right or left needle.

To enhance the overall look and minimize cuts, one pocket flap tab was omitted and sewn only at the pocket opening, which is covered by the flap. To program the machine and create a new program, a basic program was selected from the list, containing the basic parameters, and the following were added: a center knife, angled knives, a feeder, a punch, tabs for folding the pastel, left and right flap clamps, a straight pocket, a left flap laser (a photocell that reads the position of the left flap), and an on/off laser marker.

Once the pocket elements are properly aligned, the machine automatically begins the sewing process according to the programmed parameters. Creating the pocket takes approximately one minute, of which the sewing process itself takes 10 s. The time required to properly align the pocket elements before starting the process is the most significant factor. It is worth noting that the most time is spent programming the machine—up to an hour.

- Pfaff 3843 sleeve sewing machine

**Figure 16:**
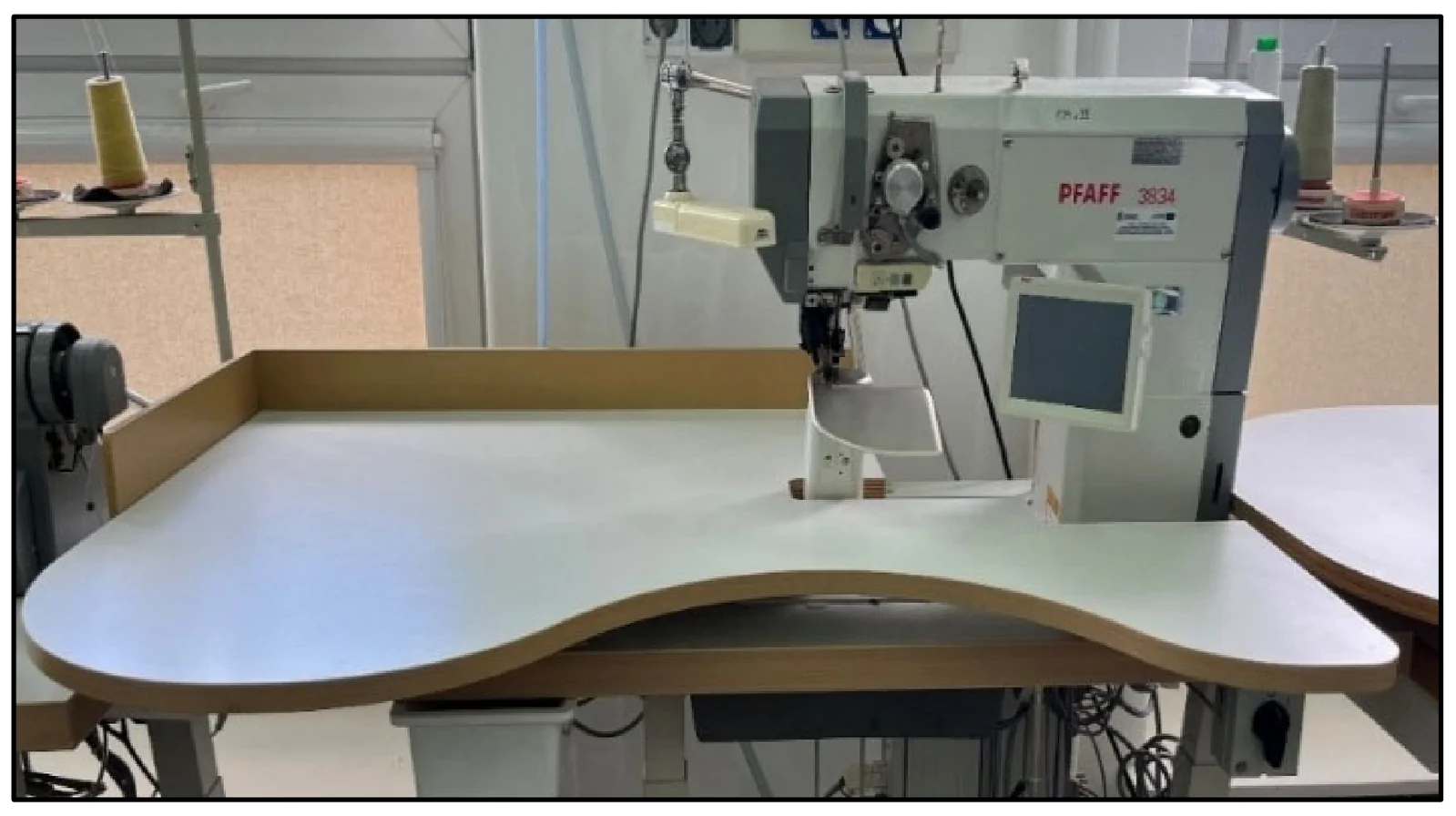
The PFAFF 3834 sleeve sewing machine.

The PFAFF 3834 sleeve sewing machine (Figure 16) is a specialized machine for sewing sleeves. It utilizes a shuttle stitch. It lacks a feed bar but is equipped with two pullers with two independent drives, allowing them to move at different speeds. The machine’s cylindrical table significantly simplifies sleeve sewing. It is equipped with an LED screen for programming.

**Figure 17:**
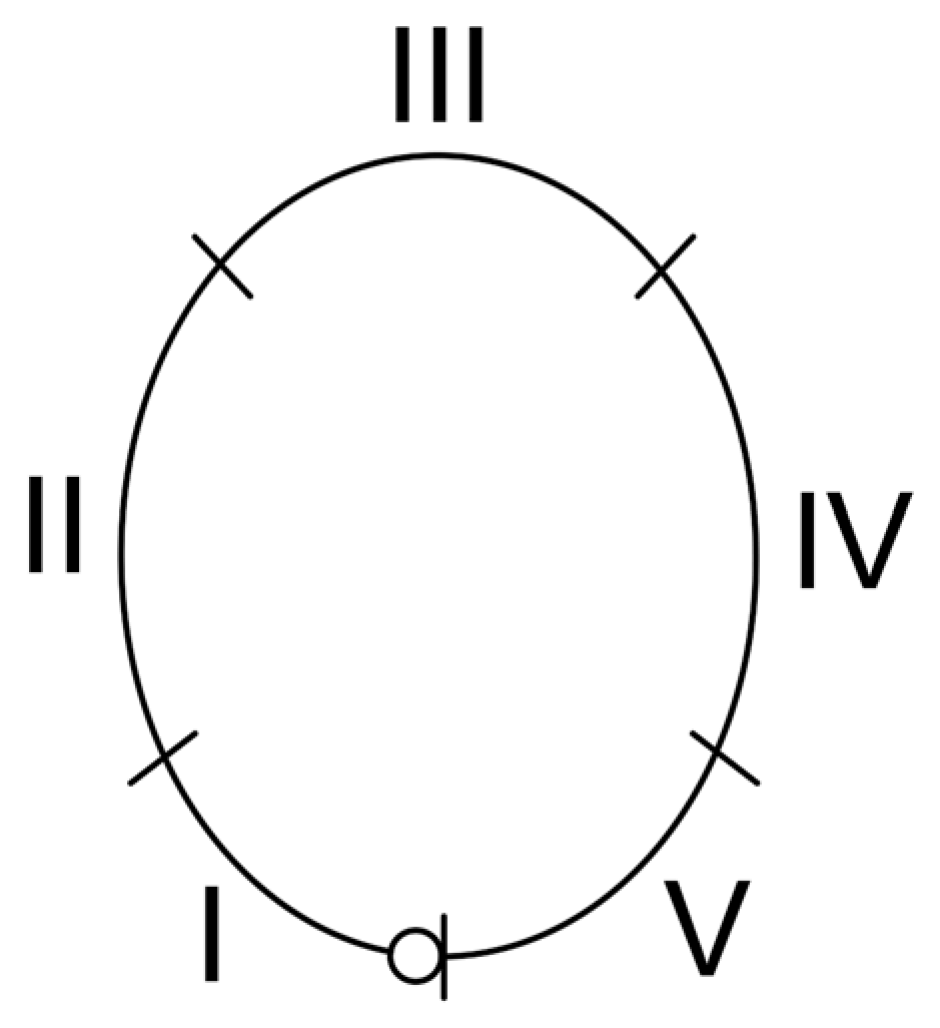
Division of sewing sections of the Pfaff 3843 specialized machine.

The operation of sewing in the sleeve and its programming is divided into five sections (Figure 17). Sections I and V are sewn in. Sections I and V are sewn flat and sections II and IV are slightly inserted, while section III is the most inserted because the sleeve head is sewn in during its execution.

- Impall JAPSEW 781X hand-stitch-imitating machine

**Figure 18:**
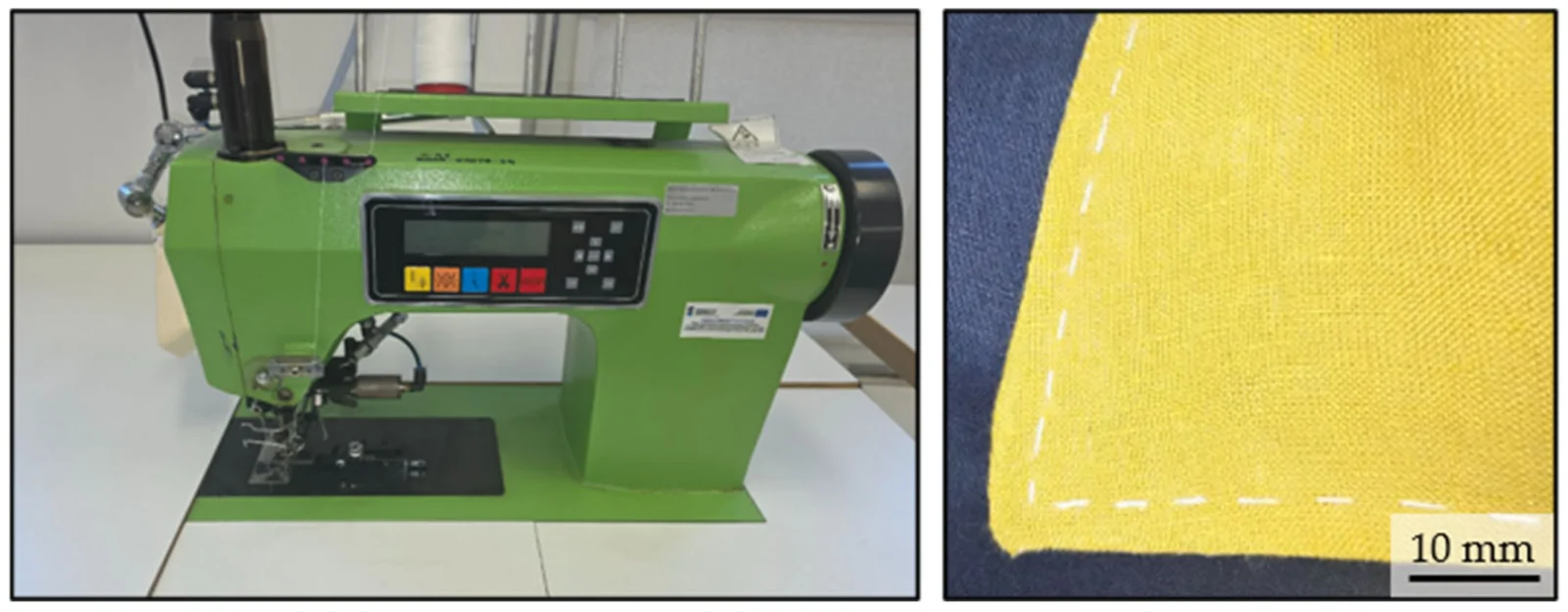
The Impall JAPSEW 781X hand-stitch machine and the stitching it produces.

A specialized machine that imitates decorative hand stitching performs a basic single-thread stitch (Figure 18). Instead of a needle, it is equipped with a special crochet hook that creates the stitch, which can reach a maximum length of 65 cm. This machine, which imitates hand stitching, is used to create decorative stitching on trousers and outerwear for both women and men.

When programming the machine, the number of stitches to initiate automatic thread trimming after three needle strokes was entered, along with the bobbin stitch length of 25 and the top stitch length of 20. Because the stitching was sewn on the underside of the collar, the longer stitching is visible on the top of the collar. The topstitch length corresponded to the stitching length on the underside of the collar, while the bottomstitch length is visible on the top of the collar.

- Dürkopp Adler 581-151 buttonhole machine

**Figure 19:**
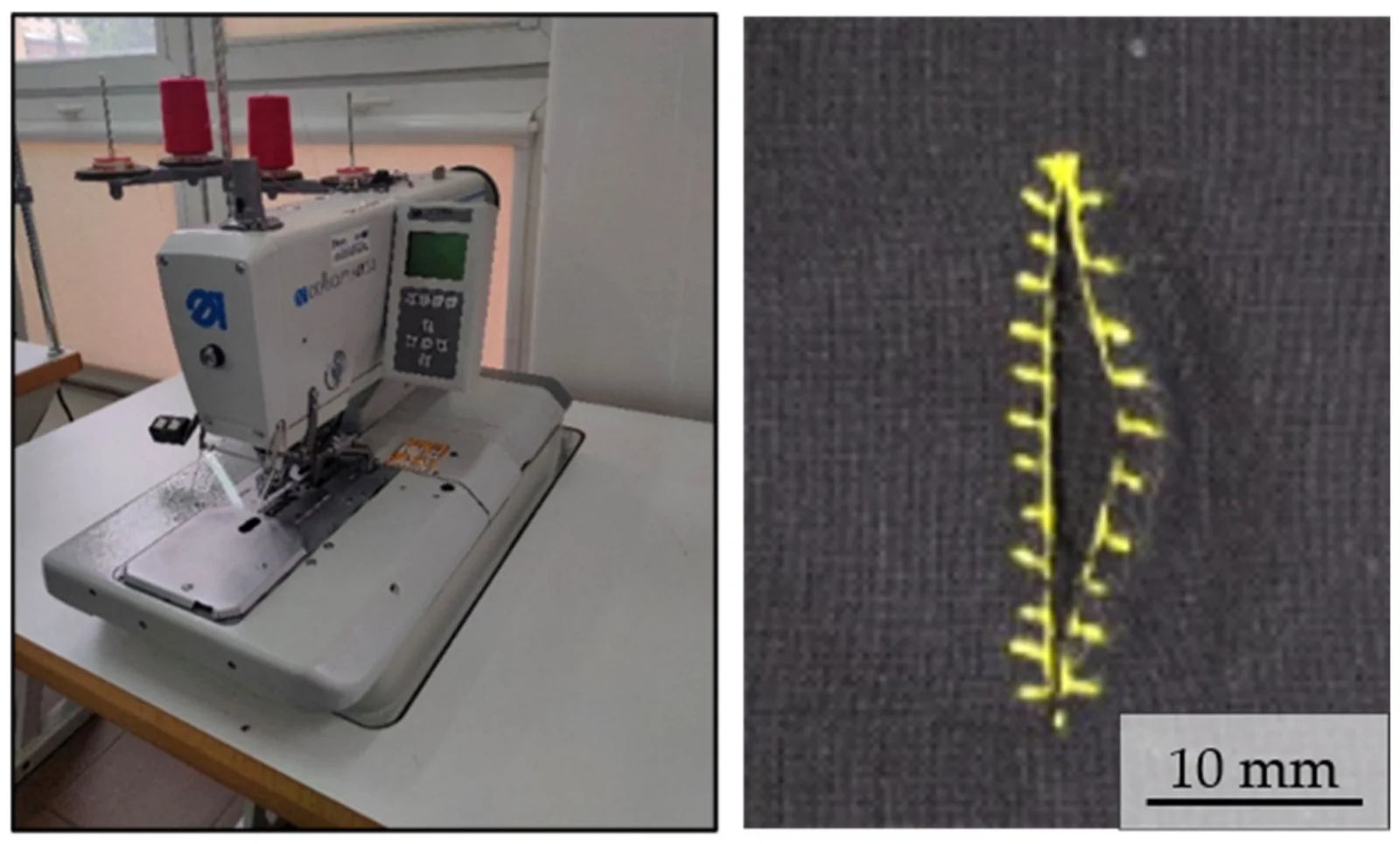
The Dürkopp Adler 581-151 buttonhole machine.

A buttonhole machine is a semi-automatic sewing machine that uses a chain stitch (Figure 19). This type of machine is designed for making buttonholes in thick materials. With the appropriate parameters, it can create a buttonhole that imitates the buttonholes made with a lingerie buttonhole machine. While the machine typically sews on the left side of the fabric, in this case, it is set to imitate a lingerie buttonhole machine that sews on the right side of the fabric [14].

When programming the machine, the appropriate buttonhole shape was selected from the program library, and the parameters were modified to meet the established requirements. The stitch length was set to 26 mm and the width to 0.5 mm so that it would most closely resemble a buttonhole that could be created on a lingerie buttonhole machine. Cutting mode was disabled, preventing the buttonhole from being automatically cut open. Each buttonhole was sewn with a different thread color, creating an additional design effect.

- PFAFF 3307 button sewing machine

**Figure 20:**
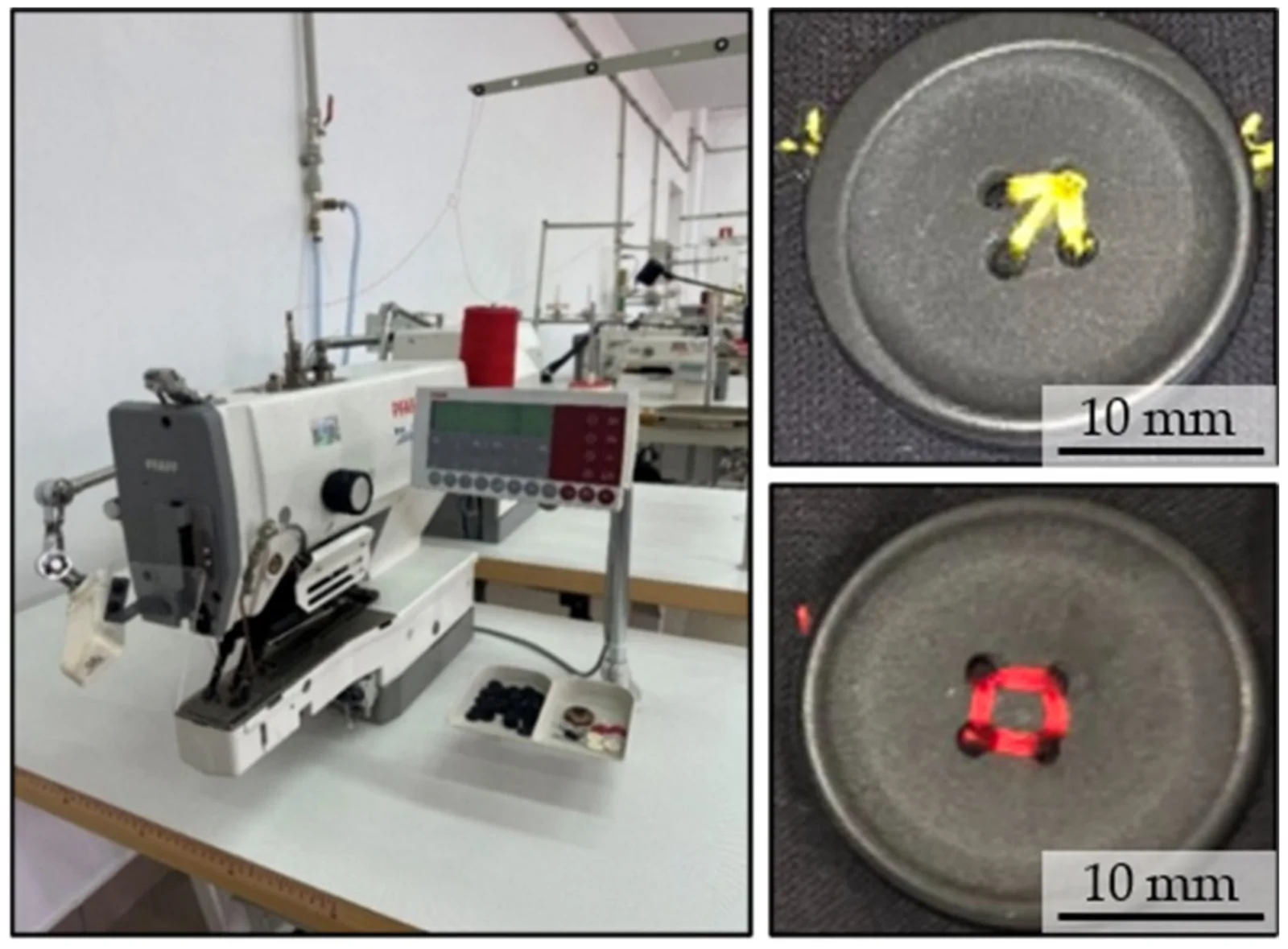
The PFAFF 3307 button sewing machine.

The Pfaff 3307 button sewing machine is a semi-automatic machine designed for sewing buttons with two, three, four, or six holes (Figure 20). The machine performs a chain stitch 14.

The programming process involves determining the needle’s coordinates on the X and Y axes so that it hits the center of the button hole. A different stitching pattern was used for each button selected for the dress. A different thread color was also used for each stitch to highlight the differences between the stitches. The final programming step involved enabling or disabling the end knot.

### 4.5. The Labor Intensity of a Personalized Women’s Dress

**Figure 21:**
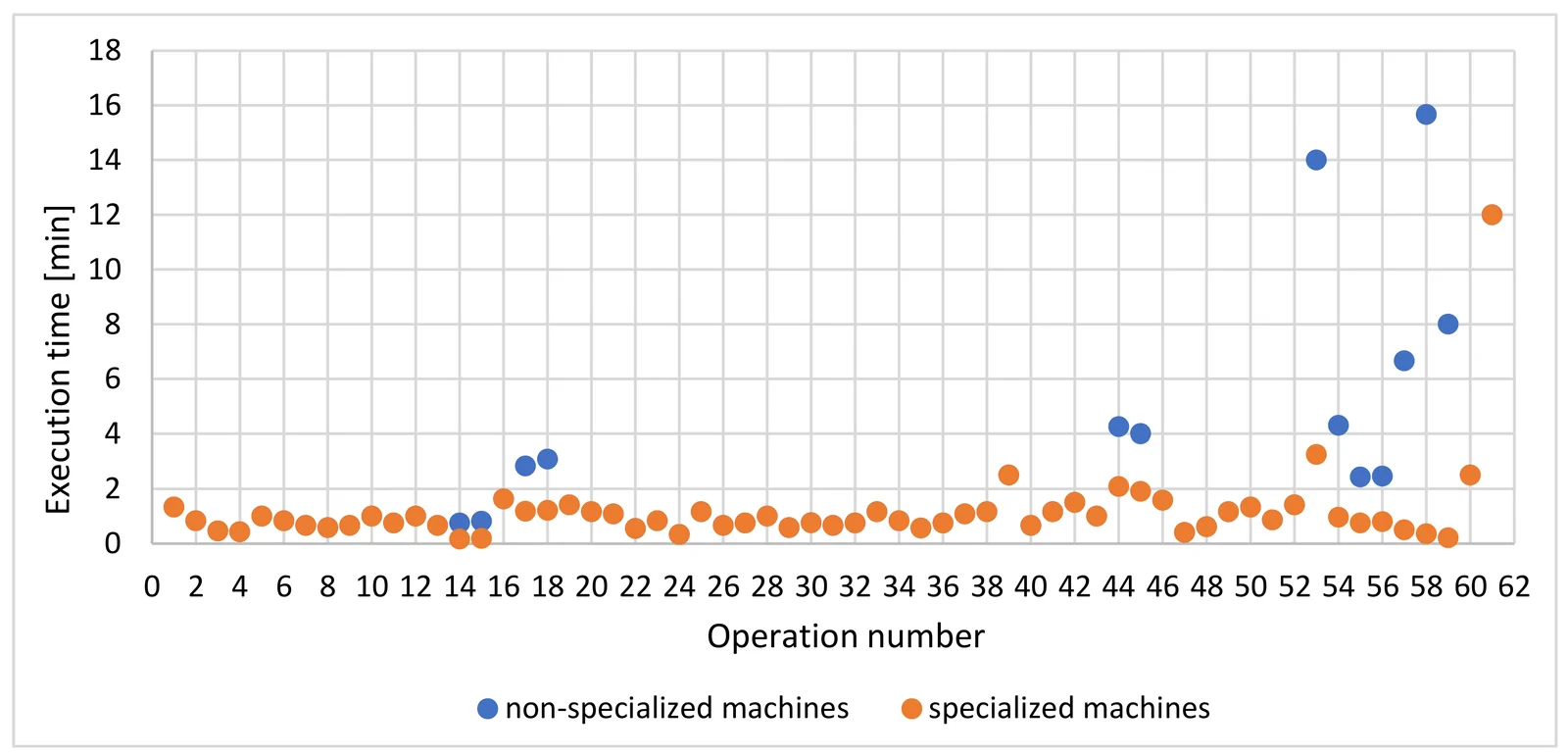
Comparison of the time it takes to make a women’s dress using specialized and non-specialized machines.

A comparative analysis of the production times for the designed, personalized women’s dress was conducted based on the sewing of two prototypes. The first was made on universal sewing machines, while the second used modern specialized machines (Figure 21). Both prototypes were sewn using 61 technological operations. The same operator made both dress prototypes. The operator had extensive experience with universal machines but limited experience with specialized, semi-automatic, and automatic machines. Processing times were measured individually for each technological operation, excluding programming times for specialized machines and rest periods. The labor intensity of the first dress prototype, made on universal sewing machines, was 126.94 min, while the second, using modern sewing machines, took 71.24 min.

Analysis of the obtained results shows a significant difference in the execution times of individual technological operations depending on the machine used or whether the operation was performed manually. For 48 technological operations, the execution times were standardized because they were performed using the same machines. However, the remaining 13 operations were performed on different machines. Where shorter operation times were observed, specialized machines, i.e., automatic or semi-automatic sewing machines, were used. The difference in the time to complete the dress prototype using modern machines and universal machines was 55.7 min.

The greatest difference can be observed for operation 58—sewing buttonholes in the front pleat. Using a buttonhole machine, the time to complete three buttonholes was 0.35 min, while in the second prototype of a women’s dress, the buttonholes were sewn by hand and took 15.66 min. A similar relationship occurs when sewing buttons to the front pleat—operation no. 59—where using a buttonhole machine took 0.21 min, while sewing them manually took 8 min. Using a semi-automatic machine saved 7.79 min. For operation 53—decorative stitching of the neck collar—using a machine imitating a hand stitch, the sewing time was 3.25 min, while performing this operation manually took 14 min. Operation 57—sewing the drawstrings—using a specialized machine took 0.5 min, while using a single-needle lockstitch machine it took 6.66 min. In the case of operation no. 54—finishing the bottom of the dress—the difference was 3.34 min and was caused by the fact that a flatbed sewing machine was used to sew the first dress prototype. In contrast, two machines were used to finish the bottom of the second prototype—a single-needle lockstitch and a three-thread overlocker—to achieve the same stitching effect for both prototype versions. For operation no. 44—sewing the right sleeve to the armhole—and operation no. 45—sewing the left sleeve to the armhole—the specialized machine reduced the completion time by 2.17 min and 2.1 min, respectively. Using the automatic machine for operation no. 17—sewing the right pocket—and operation no. 18—sewing the left pocket—reduced their completion time by 1.65 min and 1.87 min, respectively. When the automatic machine was used for operation no. 14—sewing the left flap—a difference of 0.59 min was noted. A similar relationship is visible in the case of operation 15—suturing of the right flap—where the difference was 0.63 min.

It should be noted, however, that in the case of the prototype, which was built on modern, specialized machines, the actual production time was longer, as time had to be devoted to proper programming and learning how to operate them. Programming and learning each machine took an average of 40 to 60 min. The labor-intensity analysis of the designed women’s dress did not consider the time required to program specialized machines, as in a company engaged in mass production, this process is performed once for an entire batch. The situation is different when creating a single, personalized dress tailored to the requirements of an individual user. In such a case, the need to program individual automatic, semi-automatic, and specialized machines significantly impacts the overall production time of the personalized product. Considering that preparing these machines for operation takes 40 to 60 min, the labor-intensity of a single production unit increases significantly.

The use of automatic and semi-automatic sewing machines in clothing production not only shortens the processing time but also increases the repeatability of operations and improves sewing quality. The use of modern sewing technologies fosters a deeper synergy between human labor and advanced technological solutions. Thanks to the use of specialized machines, both semi-automatic and automatic, the dress production time was reduced by 43.9% compared to universal machines.

### 4.6. Creation of a Women’s Dress Inspired by the 1960s

**Figure 22:**
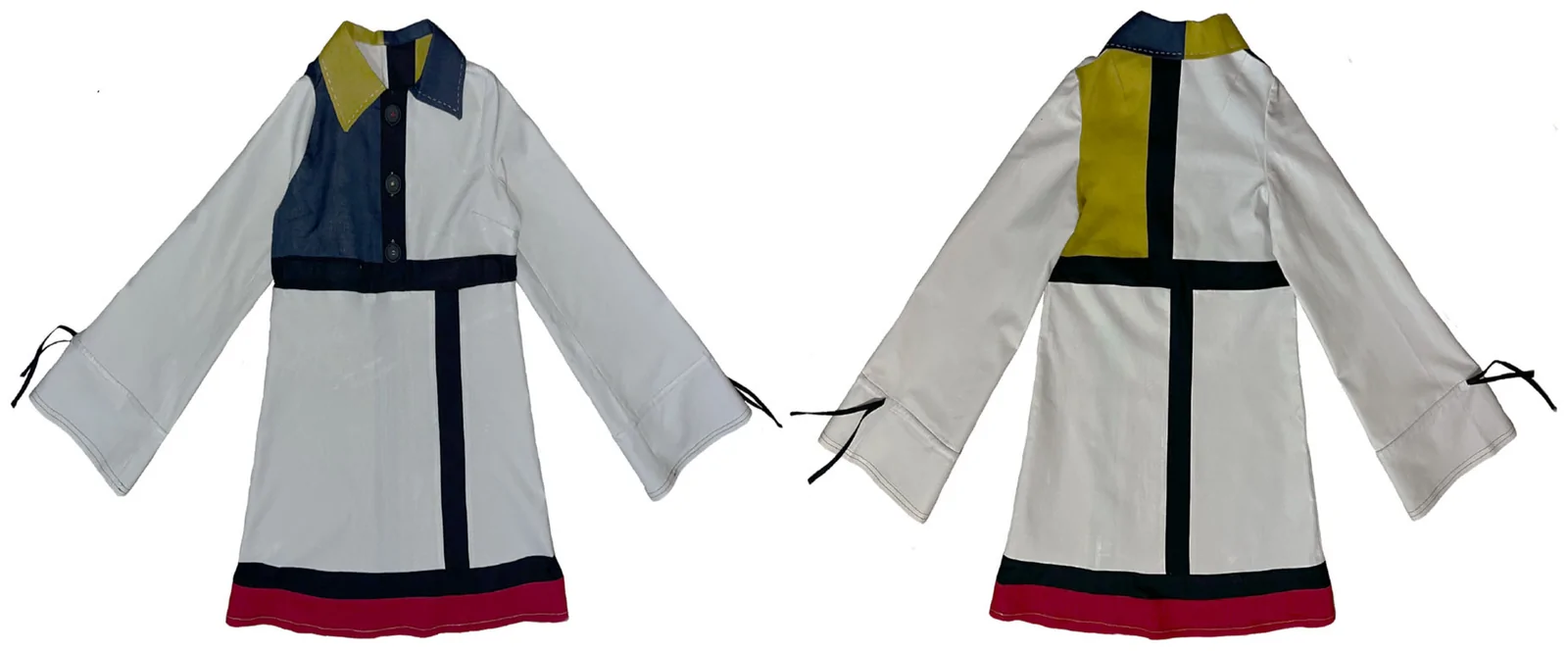
Photo of the front and back of a completed women’s dress.

Figure 22 presents photographs of a women’s dress inspired by the 1960s, made according to selected assumptions of the Industry 4.0 concept. By implementing the design in the CLO3D program and using virtual sewing capabilities, dress forms were created that allowed for the creation of a single physical prototype, reducing material waste.

## 5. Conclusions

This publication presents the potential of implementing selected Industry 4.0 concepts in the apparel sector, illustrated through the design and production process of a personalized women’s dress. The authors of the article are looking for an answer to the following question: What are the benefits and limitations of using Industry 4.0 tools in the process of designing and producing a personalized women’s dress?

To create the project, fabrics mainly made of natural fibers were used, in accordance with the principles of sustainable development. Only one linen fabric was characterized by composition: 85% linen, 10% polyester, and 5% elastane. The thermal insulation properties of the selected materials, including linen, cotton, hemp, and ramie, were analyzed.

The parameters of air permeability and thermal insulation tested on the Alambeta device confirmed that the designed women’s dress can be worn in spring.

An advantage of Industry 4.0 is the ability to produce personalized clothing products according to the individual needs and preferences of the customer. Industry 4.0 utilizes digital technologies that enable customer integration in the design and production process. To create a personalized clothing product, it was necessary to:

- Identify the customer’s needs in terms of the intended use of the clothing product (casual, business, or special occasion), preferred style, color scheme, design, material type, and functional and comfort requirements;
- Acquire anthropometric data, as a key element of personalization is the fit of the garment to the wearer’s figure; to achieve this, traditional tailoring measurements were taken for a selected female figure;
- Digital product design: based on the collected information, a clothing technologist created a model of a women’s dress in a CAD/CAM environment or CLO3D software. At this stage, a custom pattern was constructed and a virtual product visualization was created in CorelDRAW;
- Virtual prototyping: Before the physical prototype was created, a digital prototype of the dress was created on machines. The virtual fitting allowed for the identification of potential design errors and the implementation of changes without the need to sew a physical prototype.

The benefits of the Industry 4.0 concept, as exemplified by the women’s dress, include the digitization of the dress’s construction through the ability to model its forms and the virtual sewing of a product prototype in the CLO3D program, which eliminates the need to sew multiple physical prototypes and enables lower production costs and reduced material consumption, in line with the principles of sustainable development.

The premise of using modern sewing machines in relation to the Industry 4.0 concept is to deepen human–machine collaboration through a harmonious combination of human cognitive abilities with modern technological solutions. Therefore, another advantage is the use of specialized, semi-automatic, and automatic machines in one of the dress prototypes. Another significant advantage is the demonstration of the differences in labor intensity of individual technological operations between the dress prototypes produced on universal and specialized machines. Thanks to the use of special machines, the production time of the designed dress was reduced by 43.9% compared to universal machines. The analysis of the labor intensity of the production process of the designed women’s dress omitted the time required to program specialized machines, as under mass production conditions, this step is performed once for an entire series of products. The situation is different in the case of individual production, focused on creating a personalized dress according to the individual user’s requirements. In this production model, the need to prepare and program automatic, semi-automatic, and specialized machines is a significant factor influencing the overall product development time.

It is important to emphasize that Industry 4.0 also relies on automation. In the clothing sector, it is noticeable that every technological operation performed on a properly programmed sewing machine is always repeated with the same precision. With non-specialized machines, achieving this result is impossible due to the fact that the precision of the sewn element depends on the operator’s skill. The quality of sewn elements on automatic machines is always the same, whereas with non-specialized machines, the quality of the sewn elements depends on the skill and disposition of the machine operator. The latest sewing machines available on the market were selected and characterized to demonstrate their wide range of applications and possible use in the context of the Fourth Industrial Revolution.

The limitations identified in Industry 4.0 tools during the design and production of a personalized women’s dress include, above all, the high capital expenditures associated with purchasing semi-automatic and automatic sewing machines and CAD/CAM software. These barriers are particularly noticeable for small and medium-sized enterprises. Another drawback is the low flexibility associated with frequent collection changes. The clothing industry is characterized by high variability in patterns, styles, and materials, and even the smallest change in a clothing product’s design may require modifications to machine programs or replacement of their tooling. Another disadvantage is the difficulty in automating sewing processes. Woven and knitted fabrics are susceptible to deformation and stretching, and manipulation of flexible materials by robots is significantly more difficult than in many other industries. High demands on employee competencies and skills can also pose a problem. Implementing Industry 4.0 solutions in clothing companies requires specialized knowledge in automation, programming, design, and integration of digital systems. The greatest barrier negatively impacting the implementation of Industry 4.0 tools in textile companies is the limited profitability of single-item production. In the case of personalized clothing, preparatory activities play a significant role in the lead time, for example, designing the garment and creating its molds in CAD/CAM systems, as well as programming automatic and semi-automatic machines for a specific, personalized single garment.

The uniqueness of this article stems from combining analysis of the thermal insulation properties of textile materials with modern digital clothing design tools (CLO3D) and automation of production processes. Using the example of a women’s dress design, the article presents the benefits and limitations of Industry 4.0 tools in the design and production process for the clothing sector.

It can therefore be concluded that the textile sector is one of the areas in which Industry 4.0 tools can be effectively implemented, especially in the field of digital design, sewing automation, production process monitoring, data analysis and product personalization.

## Figures and Tables

**Table 1:** Characteristics of natural fiber fabrics selected for the production of a women’s dress.

Fabric Name	Composition	Enlargement ×100	Weave	Mass per Unit Area [g/m^2^]	Thickness [mm]
Linen fabric (basis)	85% linen 10% polyester 5% elastane	Right	Twill	241	0.63
		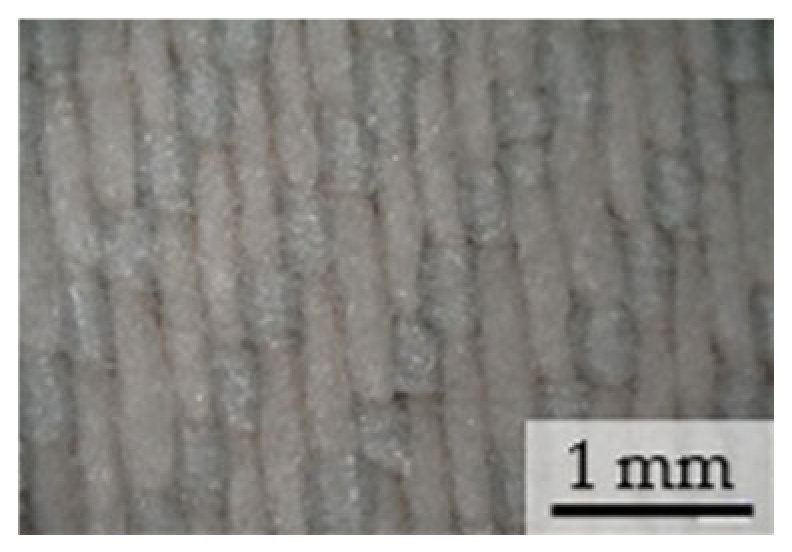			
		Left			
		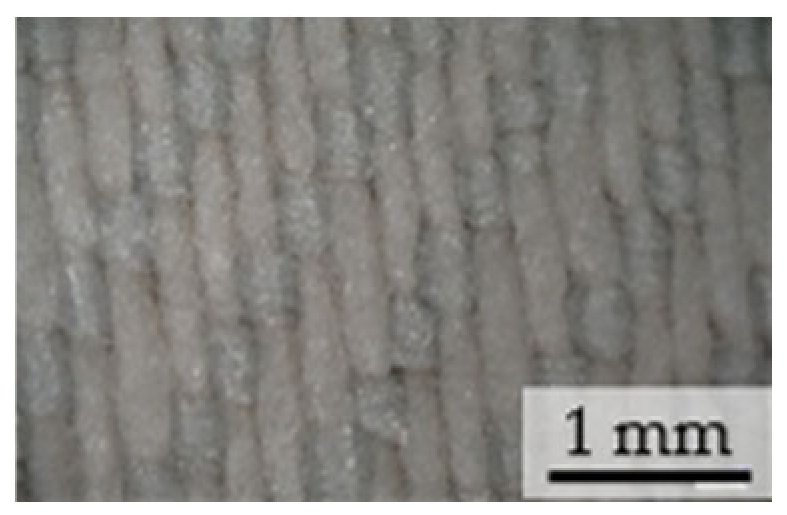			
Linen fabric	100% linen	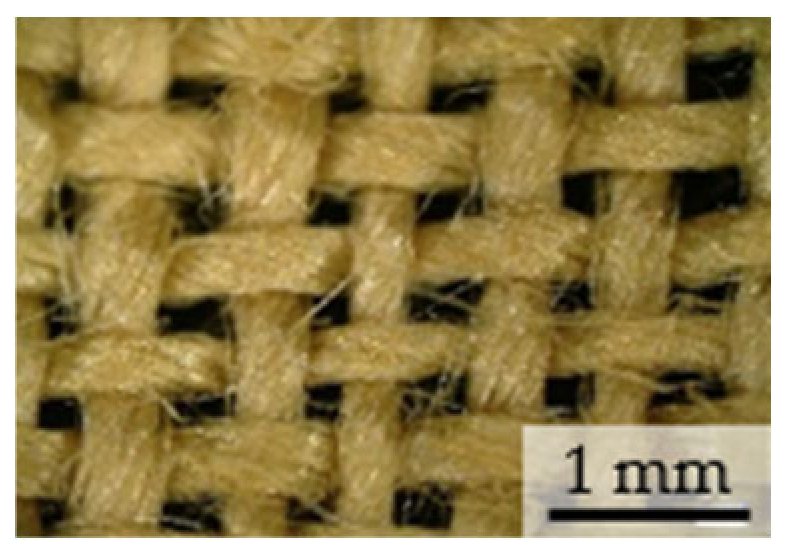	Plain	144	0.49
Hemp fabric	100% hemp	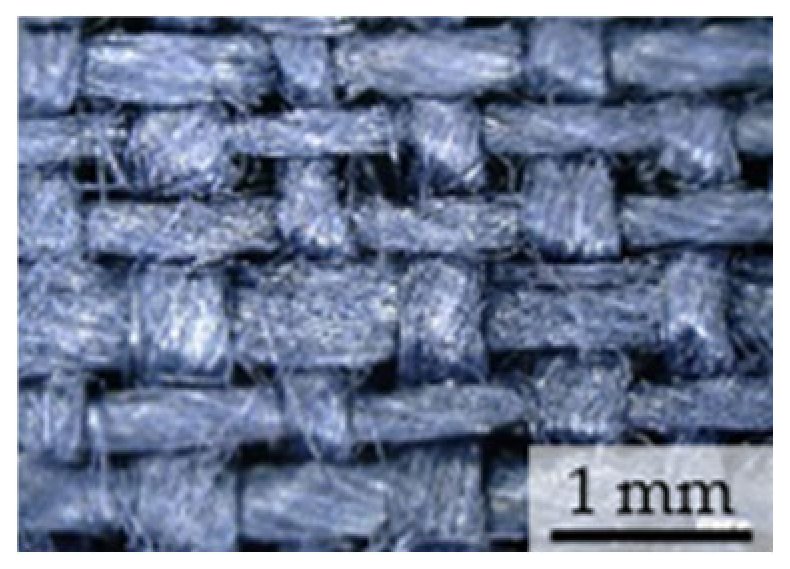	Plainy	168	0.46
Ramie fabric	100% ramie	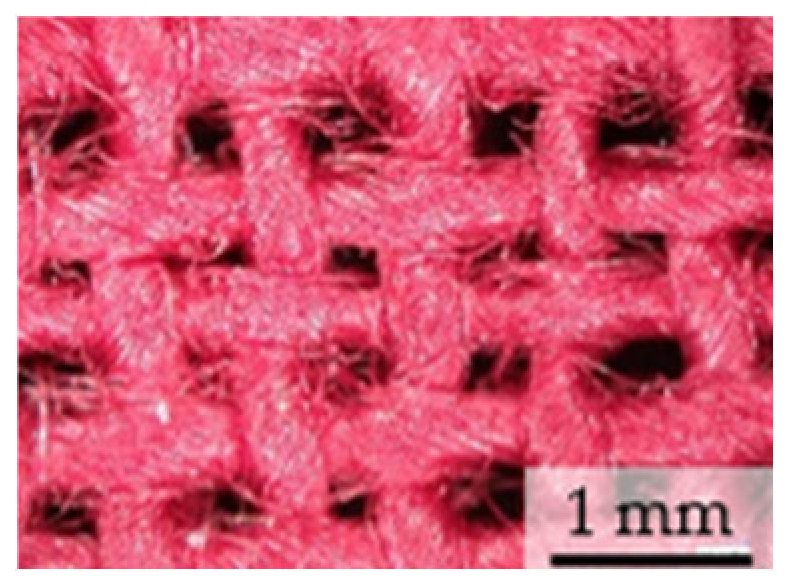	Twill	246	0.59
Cotton fabric	100% cotton	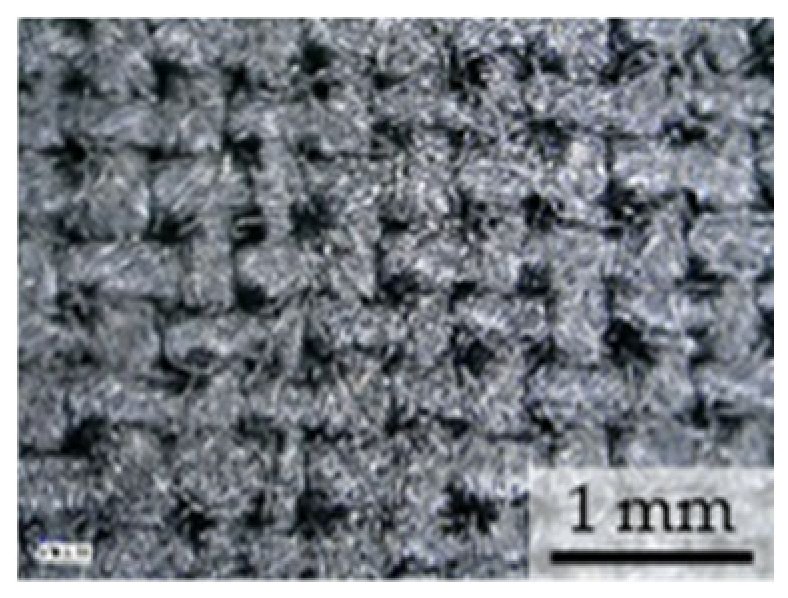	Plain	123	0.34

## Data Availability

The original contributions presented in this study are included in the article. Further inquiries can be directed to the corresponding author.
